# Posthemorrhagic hydrocephalus associates with elevated inflammation and CSF hypersecretion via activation of choroidal transporters

**DOI:** 10.1186/s12987-022-00360-w

**Published:** 2022-08-10

**Authors:** Sara Diana Lolansen, Nina Rostgaard, Dagne Barbuskaite, Tenna Capion, Markus Harboe Olsen, Nicolas H. Norager, Frederik Vilhardt, Søren Norge Andreassen, Trine L. Toft-Bertelsen, Fenghui Ye, Marianne Juhler, Richard F. Keep, Nanna MacAulay

**Affiliations:** 1 Department of Neuroscience, University of Copenhagen, Blegdamsvej 3B, DK-2200 Copenhagen, Denmark; 2 Department of Neurosurgery, The Neuroscience Centre, Copenhagen University Hospital – Rigshospitalet, Copenhagen, Denmark; 3 Department of Neuroanaesthesiology, The Neuroscience Centre, Copenhagen University Hospital – Rigshospitalet, Copenhagen, Denmark; 4 Department of Cellular and Molecular Medicine, University of Copenhagen, Copenhagen, Denmark; 5 Department of Neurosurgery, University of Michigan, Ann Arbor, USA

**Keywords:** Intraventricular hemorrhage, Subarachnoid hemorrhage, Posthemorrhagic hydrocephalus, Cerebrospinal fluid, Immune response, Biomarkers, Choroid plexus

## Abstract

**Introduction:**

Posthemorrhagic hydrocephalus (PHH) often develops following hemorrhagic events such as intraventricular hemorrhage (IVH) and subarachnoid hemorrhage (SAH). Treatment is limited to surgical diversion of the cerebrospinal fluid (CSF) since no efficient pharmacological therapies are available. This limitation follows from our incomplete knowledge of the molecular mechanisms underlying the ventriculomegaly characteristic of PHH. Here, we aimed to elucidate the molecular coupling between a hemorrhagic event and the subsequent PHH development, and reveal the inflammatory profile of the PHH pathogenesis.

**Methods:**

CSF obtained from patients with SAH was analyzed for inflammatory markers using the proximity extension assay (PEA) technique. We employed an in vivo rat model of IVH to determine ventricular size, brain water content, intracranial pressure, and CSF secretion rate, as well as for transcriptomic analysis. Ex vivo radio-isotope assays of choroid plexus transport were employed to determine the direct effect of choroidal exposure to blood and inflammatory markers, both with acutely isolated choroid plexus and after prolonged exposure obtained with viable choroid plexus kept in tissue culture conditions.

**Results:**

The rat model of IVH demonstrated PHH and associated CSF hypersecretion. The Na^+^/K^+^-ATPase activity was enhanced in choroid plexus isolated from IVH rats, but not directly stimulated by blood components. Inflammatory markers that were elevated in SAH patient CSF acted on immune receptors upregulated in IVH rat choroid plexus and caused Na^+^/K^+^/2Cl^-^ cotransporter 1 (NKCC1) hyperactivity in ex vivo experimental conditions.

**Conclusions:**

CSF hypersecretion may contribute to PHH development, likely due to hyperactivity of choroid plexus transporters. The hemorrhage-induced inflammation detected in CSF and in the choroid plexus tissue may represent the underlying pathology. Therapeutic targeting of such pathways may be employed in future treatment strategies towards PHH patients.

**Supplementary Information:**

The online version contains supplementary material available at 10.1186/s12987-022-00360-w.

## Introduction

Posthemorrhagic hydrocephalus (PHH) is a condition of progressive ventricular enlargement and elevated intracranial pressure (ICP) that develops following hemorrhagic events such as intraventricular hemorrhage (IVH) and subarachnoid hemorrhage (SAH). The condition is associated with increased morbidity and mortality [1]. PHH can develop at any age ranging from prematurity to adulthood [1, 2]. Most cases of premature PHH arise from germinal matrix bleeding [3], while adult PHH primarily develops secondarily to traumatic brain injuries, hemorrhagic strokes, or ruptured aneurysms [1]. Although PHH is a potentially devastating condition, effective prevention strategies are lacking and surgical intervention remains standard care [3–5], despite frequent complications and failures [6]. Optimization of treatment strategies for PHH requires improvement of our currently incomplete understanding of the underlying molecular pathophysiology. PHH has traditionally been attributed to impediments in cerebrospinal fluid (CSF) outflow at the arachnoid granulations initially caused by microthrombi and subsequently by inflammation and scarring [2, 7–9]. However, CSF outflow obstructions are not always discernible on diagnostic imaging [10, 11] and arachnoid granulations are absent in premature infants and in the rodents often used to model PHH [7, 12, 13].

Choroid plexus CSF hypersecretion was recently demonstrated to contribute to PHH development in an animal model of IVH [14]. CSF hypersecretion was mediated by hyperactivation of the choroidal Na^+^/K^+^/2Cl^−^ cotransporter NKCC1 [14], a key contributor to choroidal CSF secretion [15]. It can be initiated by activation of an inflammatory pathway involving Toll-like receptor 4 (TLR4) and nuclear factor-κB (NF-κB) [14] and/or by activation of the transient receptor potential vanilloid 4 (TRPV4) channel by the serum lipid lysophosphatidic acid [16]. PHH could thus originate, at least in part, from IVH-induced choroidal CSF hypersecretion [9, 14]. Such CSF hypersecretion has previously been demonstrated to underlie hydrocephalus formation in other brain pathologies such as choroid plexus hyperplasia and choroid plexus tumors [17, 18]. Hence, although CSF outflow obstructions may be causative in some cases of PHH, emerging evidence points towards an intricate interplay of pathological mechanisms including altered activity of NKCC1 and potentially other choroidal membrane transport mechanisms governing CSF secretion [2, 7, 9, 14, 16]. The exact molecular mechanisms linking hemorrhagic events to choroidal CSF hypersecretion, however, remain incompletely understood. This study therefore aimed to elucidate the molecular coupling between brain hemorrhage and PHH development and reveal the causative contribution of inflammation to the PHH pathogenesis in a rat model of PHH. Delineation of the molecular mechanisms underlying development of PHH and the potential contribution of inflammation may illuminate therapeutic targets for intervention and thereby optimize the current treatment strategy for PHH.

## Methods

### Animals

Animal experiments performed at University of Copenhagen conformed to the legislations for animal protection and care in the European Community Council Directive (2010/63/EU) and followed all ethical regulations under animal permission no. 2018-15-0201-01595 authorized by the Danish Animal Inspectorate. Animal experiments performed at University of Michigan were approved by the University of Michigan Committee on the Use and Care of Animals and followed the Guide for The Care and Use of Laboratory Animals (National Research Council, USA). Adult male Sprague Dawley rats at 9 ± 1 weeks of age (Janvier Labs, France, or Charles River Laboratories, MI, USA) were used for all experiments. The rats were housed with a 12:12 light cycle and had free access to food and water.

### Anesthesia

For the majority of the experiments, rats were anesthetized with an intraperitoneal injection of xylazine and ketamine (6 mg/ml and 60 mg/ml in sterile water, 0.17 ml per 100 g body weight, ScanVet). Pentobarbital (50 mg/kg, Leucadia) was applied for the IVH surgeries (described below) in which animals were later subjected to magnetic resonance imaging (MRI). Isoflurane (Attane vet, 1000 mg/g isoflurane, ScanVet) was applied for the MRI, as well as for the IVH surgeries in which CSF was later collected for analysis and choroid plexus collected for RNA sequencing. Isoflurane was employed using 5% (mixed with 1.8 l min^−1^ air / 0.1 l min^−1^ O_2_) to induce anesthesia in an induction chamber and 2–2.5% to maintain anesthesia through a face mask.

### IVH rat model for PHH

Rats were anesthetized and core body temperature was maintained at 37 °C by a homeothermic monitoring system (Harvard Apparatus or Yellow Springs Instrument). The femoral artery was catheterized and approximately 300 µl of arterial blood was collected. Rats were positioned in a stereotaxic frame (Kopf Instruments or Harvard Apparatus), the skull exposed with a midline incision, and a cranial burr hole drilled above the right lateral ventricle (coordinates: 0.6 mm posterior to bregma, 1.6 mm lateral to the midline, and approximately 0.6 mm ventral through the skull). 200 µl of autologous blood was injected stereotactically into the right lateral ventricle (4.5 mm ventral through the skull) with a 27-gauge needle to induce IVH [19]. The blood was injected manually or with a micro infusion pump (pump 11 elite, Harvard Apparatus) over the course of 15 min. After injection, the needle was kept in place for 5 min before retraction to prevent backflow. The skin incisions were closed with sutures and the rats were allowed to recover before returning to the housing facility. Control rats were sham-operated and received an intraventricular injection of 200 µl sterile saline. All rats were given analgesics preoperatively and once daily afterwards (carprofen 5 mg/kg, Norodyl Vet, Norbrook), in some instances supplemented with 0.4 mg/kg buprenorphine (Sandoz) preoperatively and every 12 h afterwards for 24 h.

### MRI

Anesthetized rats underwent MRI in a 9.4-Tesla MR scanner 24 h post IVH. The MRI protocol comprised a T2 fast spin-echo and T2* gradient echo sequence with the following settings: repetition time = 4000 ms and effective echo time = 60 ms for T2 weighted imaging; repetition time = 250 ms and effective echo time = 5 ms for T2* weighted imaging. Other parameters included field of view = 35 × 35 mm, matrix = 256 × 128, and slice thickness = 0.5 mm for both T2 and T2* weighted imaging. Twenty-five coronal slices were acquired to cover the entire ventricular system. MRI analysis was performed in Image J (v 1.52a) [20]. Lateral ventricle volumes were calculated from the T2 weighted images by combining the ventricle areas over all slices and multiplying by section thickness. Blood volumes were calculated from the T2* weighted images in the same manner.

### Brain water quantification

The brains were quickly removed from anesthetized rats following decapitation, and the wet weight was immediately determined on a pre-weighed porcelain evaporation beaker. Brains were afterwards homogenized with a steel pestle, placed in a pre-heated oven at 100 °C, and left to dry for 3 days. The dry brain was weighed, and the brain water content was determined in ml/g dry weight using the equation: (wet weight − dry weight)/dry weight.

### Solutions and chemicals

In vivo experiments were conducted with heated and gas-equilibrated artificial CSF (aCSF; (in mM) 127 NaCl, 2.5 KCl, 2.5 CaCl_2_, 1.3 MgSO_4_, 1 NaH_2_PO_4_, 10 glucose, 25 NaHCO_3_, pH adjusted with 95% O_2_/5% CO_2_). Ex vivo experiments (radio-isotope flux assays) were conducted in HEPES-containing aCSF (HEPES-aCSF; (in mM) 120 NaCl, 2.5 KCl, 2.5 CaCl_2_, 1.3 MgSO_4_, 1 NaH_2_PO_4_, 10 glucose, 17 Na-HEPES, adjusted to pH 7.4 with NaOH) as continuous gas-equilibration of the HCO_3_^−^ buffered aCSF is technically challenging with small quantities of isotope-containing test solutions. Initial test experiments revealed similar ^86^Rb^+^ efflux rates whether the experiments were conducted in regular gas-equilibrated aCSF or HEPES-aCSF (P = 0.155, Additional file 1, panel a). The following pharmacological agents were applied: bumetanide (B3023, Sigma-Aldrich, final concentration 20 µM) dissolved in DMSO to a stock concentration of 20 mM (D8418, Sigma-Aldrich), ouabain (O3125, Sigma-Aldrich, final concentration 2 mM) dissolved directly into HEPES-aCSF, and hemin (H9039, Sigma-Aldrich, final concentration 50 µM) dissolved in 0.7 M NaOH, C-C motif chemokine ligand 3 (CCL3), oncostatin-M (OSM), interleukin-10 (IL-10) dissolved in sterile H_2_O, and IL-6 dissolved in 10 mM HCl (CHM-343, CYT-169, CYT-465, CYT-388, Prospecbio, all final concentrations 500 ng/ml). The agents were either dissolved directly into the solution on the day of the experiment or divided into working aliquots and stored at − 20 °C to avoid repeated freeze-thaw cycles. All control solutions included the appropriate vehicle. The fluorescent dye dextran (tetramethylrhodamine isothiocyanate-dextran, MW = 150,000; T1287, Sigma-Aldrich, final concentration 0.5 mg/ml) was dissolved directly in the heated and gas-equilibrated aCSF prior to use. The incubation time of the applied pharmacological agents varied from acute exposure (bumetanide: 10 min; ouabain: 12 min; autologous blood: 5 min; hemin: ≤ 1 h) to long term exposure (16 h).

### ICP monitoring and ventriculo-cisternal perfusion

Rats were anesthetized and core body temperature was maintained at 37 °C by a homeothermic monitoring system (Harvard Apparatus). Surgical tracheotomy was performed to obtain mechanical ventilation, which was controlled with the VentElite system (Harvard Apparatus). Ventilation settings were optimized for each rat using a capnograph (Type 340, Harvard Apparatus) and a pulse oximeter (MouseOx^®^ Plus, Starr Life Sciences) to ensure physiologically relevant blood gas levels. Anesthetized and ventilated rats were positioned in a stereotactic frame (Harvard Apparatus) and the skull exposed through a midline incision. A 4.5 mm brain infusion cannula (Brain infusion kit 2, Alzet) was inserted into the right lateral ventricle through the cranial burr hole made during the PHH procedure (to be employed for the subsequent ventriculo-cisternal perfusion described below). A cranial window (approximately 3.6 mm in diameter) was drilled on the contralateral side of the skull and an epidural probe (PlasticsOne, C313G) was lowered and secured with dental resin cement (Panavia SA Cement, Kuraray Noritake Dental Inc.). The ICP cannula was pre-filled with HEPES-aCSF and connected to a pressure transducer (APT300) and transducer amplifier module TAM-A (both from Hugo Sachs Elektronik). The pressure signal was recorded with a 1 kHz sampling rate using BDAS Basic Data Acquisition Software (Harvard Apparatus, Hugo Sachs Elektronik). To assure a continuous fluid column between the dura and the epidural probe, approximately 10 µl of HEPES-aCSF was injected through the epidural probe after which jugular compression was applied to confirm proper ICP recording. After observing a stable ICP signal, the measurement was continued for 15 min and the average ICP was determined. The recording and analysis of ICP signals were performed in a blinded manner. The ventriculo-cisternal perfusion procedure [15, 21–23] was initiated immediately after completion of the ICP monitoring. The perfusion solution entering the brain infusion cannula (heated and gas-equilibrated aCSF containing 0.5 mg/ml TRITC-dextran) was heated to 37 °C by an inline heater (SF-28, Warner Instruments). After separation of the neck muscle layers, cisterna magna was punctured by insertion of a glass capillary (30–0067, Harvard Apparatus pulled by a Brown Micropipette puller, Model P-97, Sutter Instruments) at a 5° angle. The following 2 h, aCSF was continuously infused into the right lateral ventricle at a rate of 9 µl/min using a peristaltic pump, while CSF was collected from cisterna magna at 5 min intervals from a second glass capillary (30-0065, Harvard Apparatus) inserted into the first capillary. The fluorescence of the collected CSF samples was measured using the Synergy™ Neo2 Multi-mode Microplate Reader (545 nm, BioTek Instruments). The CSF production rate was obtained in the time interval between 60 and 120 min in a blinded manner and calculated from the equation [21]:

$${\text{V}}_{{\text{p}}} = {\text{r}}_{{\text{i}}} \cdot\left( {{\text{C}}_{{\text{i}}} - {\text{C}}_{{\text{o}}} } \right)/{\text{C}}_{{\text{o}}}$$

where V_p_ = CSF production rate (µl/min), r_i_ = infusion rate (µl/min), C_i_ = fluorescence of inflow solution (a.u.), C_o_ = fluorescence of outflow solution (a.u.).

### RNA-sequencing

Isolated rat choroid plexus (lateral and 4th) obtained from rats undergoing the IVH procedure or saline-injected sham operated animals were pooled according to experimental condition, (n = 4 of each) and stored in RNAlater (R0901, Sigma-Aldrich) at − 80 °C. RNA extraction and library preparation were performed by Novogene Company Limited, UK with NEB Next^®^ Ultra™ RNA Library Prep Kit (New England Biolabs) prior to RNA sequencing (RNAseq) (paired-end 150 bp, with 12 Gb output) on an Illumina NovaSeq 6000 (Illumina). The 150 base paired-end reads were mapped to reference genome (Rattus norvegicus Rnor_6.0) using Spliced Transcripts Alignment to a Reference (STAR) RNA-seq aligner (v 2.7.2a) [24]. The mapped alignments by STAR were normalized to transcripts per million (TPM) with RSEM (v 1.3.3) [25]. Gene information was gathered with mygene (v3.1.0) python library [26–28], from which gene symbol, alias, and Gene Ontology (GO) terms [29–31] were collected. Program settings for library building, mapping, and all scripts for gene annotation and analysis are available at https://github.com/Sorennorge/posthemorrhagic_hydrocephalus. We defined differentially expressed genes as ≥ 20% different and with a TPM difference of ≥ 1 TPM. The obtained data are divided into those with TPM ≥ 0.5 for both conditions (control and PHH) and those with TPM < 0.5 in one of the two conditions with a subdivision, when required, illustrating those without detection (0 TPM) in one of the two conditions (see separate Excel sheets in the Additional files 3, 8, 9). Genes that were expressed at < 0.5 TPM in both conditions were excluded from the analysis. The genes encoding proteins involved in the immune response was gathered based on GO terms ‘Inflammation’, ‘inflammatory’, ‘Immune’, ‘Cytokine’, ‘Chemokine’, ‘Interleukin’, ‘Lymphokine’, ‘Interferon’, ‘Toll-like receptor’, ‘Tumor necrosis factor’, ‘Transforming growth factor’, ‘Cyclooxygenase’, ‘Oxidative stress’, ‘Epiplexus’, and ‘Kolmer’. The immune list was manually curated and divided into receptors and non-receptors.

### Choroid plexus isolation and cell culture

Isolated rat brains were immersed in ice-cold HEPES-aCSF for 10 min before isolation of the lateral choroid plexus. In a subset of experiments, the acutely isolated lateral choroid plexus was placed in Dulbecco’s Modified Eagle’s Medium **(**DMEM1965, Gibco) supplemented with penicillin-streptomycin (100 U/ml, 100 µg/ml, Gibco), rat epidermal growth factor (10 ng/ml, SRP3238, Sigma-Aldrich), and fetal bovine serum (10%, 04-007-1A, Biological Industries), and incubated at 37 °C, 5% CO_2_, for 16 or 24 h prior to initiation of subsequent experiments.

### ^86^Rb^+^ influx and efflux

The isolated lateral choroid plexus was placed in 37 °C HEPES-aCSF for a 10-min recovery period followed by 2 min (influx) or 5–10 min (efflux) of incubation in an isotope solution containing: rubidium (^86^Rb^+^) (1 µCi/ml, 022-105721-00321-0001, POLATOM) and ^3^H-mannitol (4 µCi/ml, NET101, Perkin Elmer). ^86^Rb^+^ acts as a congener for K^+^ and therefore readily enters the cells, whereas ^3^H-mannitol remains on the outside and serves as an extracellular marker [32]. For influx, the choroid plexus was subsequently rinsed in ice-cold isotope-free HEPES-aCSF containing 2 mM ouabain, 20 µM bumetanide, and 1 mM BaCl_2_ (to prevent efflux of intracellular ^86^Rb^+^ during the washing procedure), followed by transfer into scintillation vials containing 100 µl Solvable (6NE9100, Perkin Elmer). For efflux, the choroid plexus was swiftly rinsed in 37 °C isotope-free HEPES-aCSF followed by transfer into new wells containing 37 °C isotope-free HEPES-aCSF at 5–20 s intervals. For every time point, 200 µl of surrounding HEPES-aCSF was subsequently collected and placed into scintillation vials. At the end of the experiment, the choroid plexus was placed into a scintillation vial containing 200 µl Solvable. For both influx and efflux, the choroid plexus was dissolved completely before the isotope content was determined in 2 ml Ultima Gold™ XR scintillation liquid (6013119, Perkin Elmer) using the Tri-Carb 2900TR Liquid Scintillation Analyzer (Packard). The ^86^Rb^+^ activity was corrected for extracellular background using ^3^H-mannitol [32]. Efflux data are shown as the natural logarithm of the ^86^Rb^+^ activity at each time point (A_T_) normalized to the initial ^86^Rb^+^ activity (A_0_) as a function of time. The slope from linear regression analysis was used to determine the ^86^Rb^+^ efflux rate constant (min^−1^) [15, 32].

### Calcein-AM survival assay

Calcein acetoxymethyl (calcein-AM) staining was employed to verify choroid plexus epithelial cell viability [33]. Viable cells contain active intracellular esterases that cleave the AM group from the calcein, resulting in a bright fluorescent signal from these cells. Choroid plexus was incubated in aCSF containing calcein-AM (16.7 µM, C3100MP, Invitrogen) for 10 min at room temperature and transferred to microscope slides for microscopy. Images were acquired using Zeiss Axioplan 2 microscope equipped with epifluorescence and interference filters with a 702 moni AxioCam and using Zeiss Zen Black software.

### Electron microscopy and light microscopy

Choroid plexus, acutely isolated or after up to 24 h of tissue culture, was fixed in 2% glutaraldehyde in phosphate buffer, pH 7.2 before standard Epon embedding for conventional transmission electron microscopy (TEM). TEM sections (60 nm) were examined in a CM 100 microscope and images acquired with an Olympus Veleta camera at a resolution of 2048 × 2048. Thicker sections were stained with toluidine blue for light microscopical examination.

### Western blotting

Isolated lateral choroid plexus was lysed in RIPA buffer (in mM: 150 NaCl, 50 Tris pH 8.0, 5 EDTA, 0.5% sodium deoxycholate, 0.1% SDS and 1% Triton X-100) supplemented with the protease inhibitors pefabloc (0.4 mM, Sigma-Aldrich) and leupeptin (8 µM, Sigma-Aldrich), sonicated (70% power for 10 s intervals, Sonopuls, Bandelin), and centrifuged at 10,000 × *g* for 5 min at 4 °C prior to protein concentration determination with the DC Protein Assay (Bio-Rad). 10–20 µg protein was loaded on precast SDS-PAGE gels (4–20% Criterion TGX, Bio-Rad) and transferred using immobilon-FL membranes (Merck Millipore). Primary and secondary antibodies were diluted 1:1 in Odyssey blocking buffer (LI-COR): PBS-T. Primary antibodies: anti-GAPDH; AB2302 (Millipore, 1:5000), anti-NKCC1; S022D (MRC PPU Reagents, 2 µg/ml), anti-Na^+^/K^+^-ATPase α1; a6F (DSHB, 1:60). Secondary antibodies, all 1:10,000; IRDye 680RD donkey anti-chicken (LI-COR, P/N 925-68075), IRDye 800CW goat anti-mouse (LI-COR, P/N 926-32210), rabbit anti-sheep (Thermofisher, SA5-10060). Images were obtained by the Odyssey CLx imaging system and analyzed by Image Studio ver. 5.2 (LI-COR).

### Rat CSF sampling

Anesthetized rats were positioned in a stereotactic frame (Harvard Apparatus) and the cisterna magna quickly (within 10 min) punctured in the same manner as described for ventriculo-cisternal perfusion (see above). The CSF was collected in polypropylene tubes (Cat. No. 72.730.006, Sarstedt) and immediately centrifuged at 2000 × *g* for 10 min at 4 °C prior to aliquoting and storage at − 80 °C until use to avoid repeated freeze thaw cycles.

### Patient samples

CSF samples were collected from 12 patients (median age: 68 years, range 56–73, 1 M/11 F) with acute SAH admitted and treated for the condition at the Department of Neurosurgery at Rigshospitalet, Copenhagen, Denmark. CSF samples were obtained within 24 h of ictus (8/12) or as soon as possible hereafter (4/12) through an external ventricular drain (EVD) inserted on clinical indications. Patients were selected for the present study if they were subsequently diagnosed with PHH and received a permanent ventriculo-peritoneal shunt because of continued need for CSF diversion. As control subjects, 13 patients undergoing preventive surgery for unruptured aneurysms (vascular clipping) were enrolled (median age: 68 y, range 41–73, 5 M/8 F), and CSF was collected from the basal cisterns during surgery prior to clipping of the aneurysm. Written informed consent was obtained from all patients or next of kin depending on the capacity of the patients and the study was approved by the Ethics committee of the Capital Region of Denmark (H-19001474 and H-17011472/69197) and the Danish Data Protection Agency (P-2019-773).

### CSF analysis

Rat and human CSF samples were analyzed for presence of 92 different inflammatory markers (Inflammation panel, Art. No. 95302, https://www.olink.com/products-services/target/inflammation/) by BioXpedia A/S (Aarhus, Denmark) using the proximity extension assay (PEA) technique (Olink Bioscience). The PEA technique enables simultaneous detection of 92 inflammatory markers in a CSF volume of only 1 µl, thus providing a high throughput while maintaining excellent sensitivity and specificity [34]. The detection of each inflammatory marker is accomplished using paired antibodies with attached oligonucleotides, which hybridize and form a detectable PCR sequence upon presence of the inflammatory marker. The PCR sequence is then amplified and quantified by real-time qPCR. The PEA output is given in normalized protein expression (NPX) values, an arbitrary unit on log_2_ scale. The limit of detection (LOD) was calculated for each inflammatory marker as the background signal plus three times the standard deviation of the inflammatory marker. Inflammatory markers were excluded from further analysis if  > 35% of the samples were below the LOD and no preferential presence in any study group was detected. This criterion led to the exclusion of 34 inflammatory markers for the human CSF samples and 78 inflammatory markers for the rat CSF samples. The remaining inflammatory markers (58 for humans, 14 for rats) were included in the statistical analysis. For normally distributed data, an unpaired two-tailed t-test was conducted and Welch’s correction was applied if variances were unequal. For non-normally distributed data, a Mann-Whitney U test was conducted. *P* values were corrected for multiple comparisons using the Bonferroni method [35]. A flow diagram of the CSF analysis process is provided in Additional file 2.

### Data analysis

The number of animals chosen for the in vivo experiments was determined as earlier described [15], whereas the remaining rat experiments were considered exploratory and designed according to the principles of the 3Rs (Replacement, Reduction and Refinement) to limit our rat usage. The number of patients enrolled for CSF analysis (12/13) was employed according to [36]. Data analysis and statistical significance was obtained with Prism, v.9 (GraphPad). Data are presented as mean ± SD with the statistical test indicated in figure legends.

## Results

### IVH leads to development of PHH

**Fig. 1:**
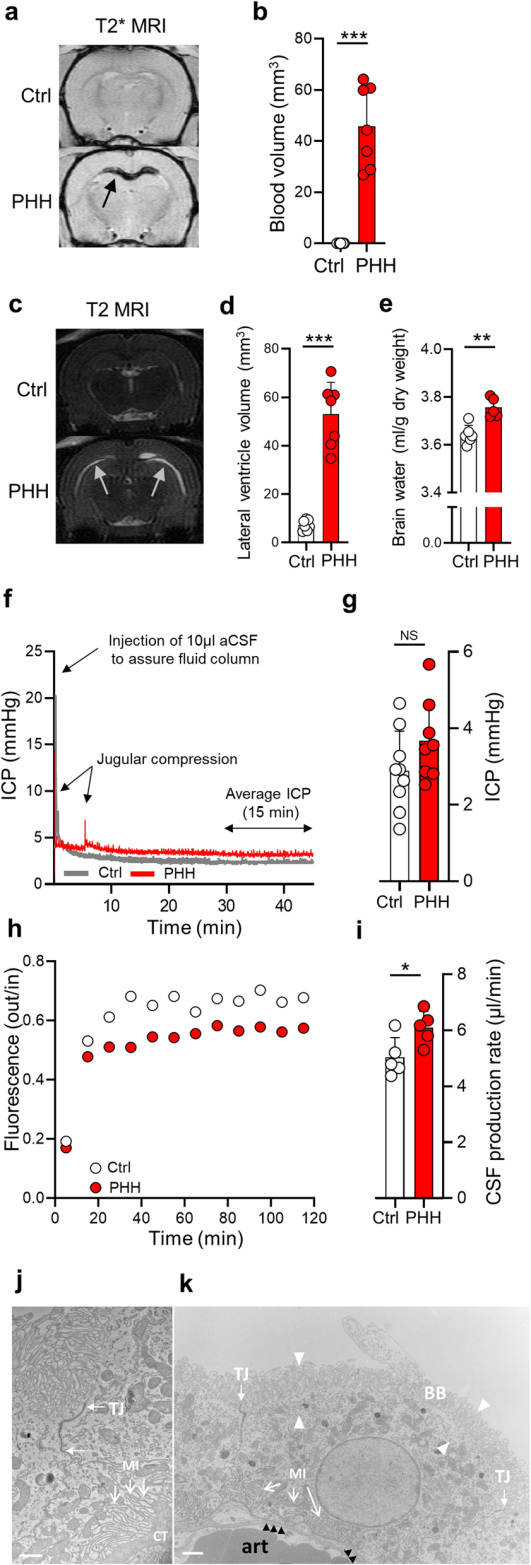
PHH is associated with excessive brain water accumulation and CSF hypersecretion.** a** Representative T2* weighted MRI rat brain sections 24 h after injection of 200 µl sterile saline (ctrl) or autologous blood (PHH) into the right lateral ventricle. The dark region (black arrow) indicates presence of blood. **b** Blood volume quantification from MRI sections 24 h after injection of saline (ctrl, n = 7) or autologous blood (PHH, n = 7). **c** Representative T2 weighted MRI rat brain sections 24 h after injection of saline (ctrl) or autologous blood (PHH) into the right lateral ventricle. The bright regions (white arrows) indicate presence of CSF within the lateral ventricles. **d** Lateral ventricle volume quantification from MRI sections 24 h after injection of saline (ctrl, n = 7) or autologous blood (PHH, n = 7). **e** Brain water content quantified from control rats (n = 6) and PHH rats (n = 5) 24 h post IVH using the wet and dry brain weight. **f** Representative ICP traces for one control rat and one PHH rat 24 h post IVH. **g** Average ICP in control (n = 9) and PHH rats (n = 8) quantified from a stable 15 min time period. **h** Representative time course traces of the fluorescence ratio of dextran (outflow/inflow) during ventriculo-cisternal perfusion of one control rat and one PHH rat 24 h post IVH. **i** Average CSF production rate in control (n = 5) and PHH rats (n = 5) quantified from the fluorescence ratio of dextran after obtaining a stable baseline (at 60 min). Electron microscopy of choroid plexus isolated from rats subjected to control saline treatment (**j**) or IVH (**k**) for 24 h. Saline-treated choroid plexus shows the typical well-developed brush border, tight junctions (TJ), and extensive basolateral membrane infoldings (MI) towards the underlying basal membrane and connective tissue (CT). Choroid plexus obtained from IVH rats (**k**) displayed intact differentiated traits, also in the organelle-sparse cortical zone (in between arrowheads). Black arrows point to a thin rim of connective tissue separating the epithelium from an underlying arteriole (art). Scale bars, 1 mm. Error bars represent standard deviation and statistical significance was tested with an unpaired two-tailed t-test. *P < 0.05, **P < 0.01, ***P < 0.001, *NS* not significant

To produce an experimental model of PHH with which to elucidate the molecular mechanisms underlying the hydrocephalus often associated with brain hemorrhage in patients, we mimicked the pathological condition by delivery of autologous blood (obtained from the femoral artery) into the right lateral ventricle of anesthetized rats. Ventricular blood deposits were demonstrated in these rats 24 h later by T2* weighted MR scans (blood volume of 45.8 ± 15.8 mm^3^, n = 7), which was absent in control rats undergone sham surgery on the femoral artery and intraventricular delivery of saline (*P* < 0.001, Fig. 1a, b). Quantification of the ventricular volume in these same animals, obtained by T2 weighted MR scans, demonstrated enlarged lateral ventricles in the IVH rats (53.0 ± 13.2 mm^3^, n = 7) compared to the control rats (7.28 ± 2.13 mm^3^, n = 7, *P* < 0.001, Fig. 1c, d), thus demonstrating that our rodent IVH experimental model develops PHH. To verify that the observed ventriculomegaly was associated with enhanced brain water accumulation, we obtained the water content of the rat brains 24 h post IVH with the wet-dry technique [37]. The brain water content of PHH rats was significantly higher than that obtained in control rats (3.76 ± 0.04 ml/g dry weight vs. 3.64 ± 0.04 ml/g dry weight, n = 5–6, *P* < 0.01, Fig. 1e). Experimental IVH in rats is thus associated with ventricular enlargement and excessive brain water accumulation. To determine whether rodent PHH associates with an elevated ICP, as observed clinically [1] and indicative of excessive brain water accumulation, we measured the ICP of anesthetized and ventilated PHH rats 24 h post IVH (Fig. 1f). Although a tendency was apparent, the ICP was not significantly elevated in PHH rats (3.67 ± 1.05 mmHg, n = 8) compared to controls rats (2.92 ± 1.04 mmHg, n = 9, *P* = 0.14, Fig. 1g). Hence, at the early time point of 24 h post IVH, the PHH-mediated elevated brain water content and enlarged ventricles in our rodent model of PHH do not suffice to translate to elevated ICP.

### PHH associates with CSF hypersecretion

To determine whether the excessive brain water accumulation was associated with CSF hypersecretion, CSF production rate was quantified in anesthetized and ventilated PHH rats 24 h post IVH using the ventriculo-cisternal perfusion technique [15]. Here, the ventricular system of the rats was continuously perfused by delivery of heated and gas-equilibrated aCSF containing fluorescent dextran into the right lateral ventricle with simultaneous CSF sampling through a cisterna magna puncture. The dilution of the fluorescence during its path through the ventricular system arises from newly formed CSF. Thus, lower fluorescence content in the cisternal samples corresponds to a higher degree of dilution and thus a higher CSF production rate (Fig. 1h). The fluorescence dilution was higher in PHH rats than in their control counterparts (Fig. 1h) and the CSF secretion rate therefore elevated in the PHH rats (6.10 ± 0.60 µl/min vs. 5.04 ± 0.70 µl/min, n = 5, *P* < 0.05, Fig. 1i). The CSF hypersecretion did not arise due to hemorrhage-induced breakdown of the blood-CSF barrier as the structural integrity of choroid plexus was apparently retained following the hemorrhagic event, as evidenced by electron microscopy (Fig. 1j, k). IVH-mediated PHH is thus associated with CSF hypersecretion across an intact choroid plexus.

### Choroid plexus transporters are upregulated in PHH rats

As CSF hypersecretion could arise from IVH-mediated upregulation of choroidal transport mechanisms, we determined the transcriptional abundance of select membrane transport proteins involved in CSF secretion. RNAseq on choroid plexus obtained from PHH rats and control rats 24 h post IVH revealed transcription of 13,884 genes in the choroid plexus. Of the transcripts detected at ≥ 0.5 TPM, 5,944 were considered differentially transcribed between the two experimental groups, with the vast majority (96%) being upregulated in PHH rats compared to their control counter-parts (Additional file 3). A manual search of this gene list revealed several transport proteins implicated in CSF secretion [38, 39] as upregulated in the choroid plexus of PHH rats compared to control rats (Table 1): Aquaporin 1 (AQP1), a water channel located at the luminal membrane of the choroid plexus, doubled in transcriptional abundance. Transcripts encoding the Na^+^/K^+^-ATPase α1 subunit, the dominant catalytic Na^+^/K^+^-ATPase subunit in the choroid plexus, and NKCC1 (both expressed on the luminal membrane of choroid plexus) displayed ~ 30% upregulation. The accessory β3 subunit of the Na^+^/K^+^-ATPase was similarly augmented as was the basolaterally-located Na^+^-driven chloride bicarbonate exchanger, NCBE. Hence, various choroidal transport proteins implicated in CSF secretion demonstrate an upregulation at the transcriptional level in the setting of PHH in rats.

**Table 1:** Transport proteins implicated in CSF secretion. Choroidal RNAseq data are given in transcript per million (TPM) for control rats and PHH rats (n = 4 of each)

Gene	Alias	Description	Control (TPM)	PHH (TPM)	Log_2_FC	Upregulated (%)
ATP1A1	NKAα1	Na^+^/K^+^-ATPase α1	392	534	0.44	36
ATP1B3	NKAβ3	Na^+^/K^+^-ATPase β3	196	283	0.53	44
SLC12A2	NKCC1	Na^+^/K^+^/2Cl^−^ cotransporter	200	257	0.36	29
SLC4A10	NCBE	Na^+^/HCO_3_^−^ cotransporter	245	306	0.32	25
AQP1	AQP1	Aquaporin 1	482	969	1.01	101

Changes at the transcriptional level are indicated by the log_2_ fold-change (Log_2_FC) and the upregulation in percent

### PHH associates with Na^+^/K^+^-ATPase hyperactivity

**Fig. 2:**
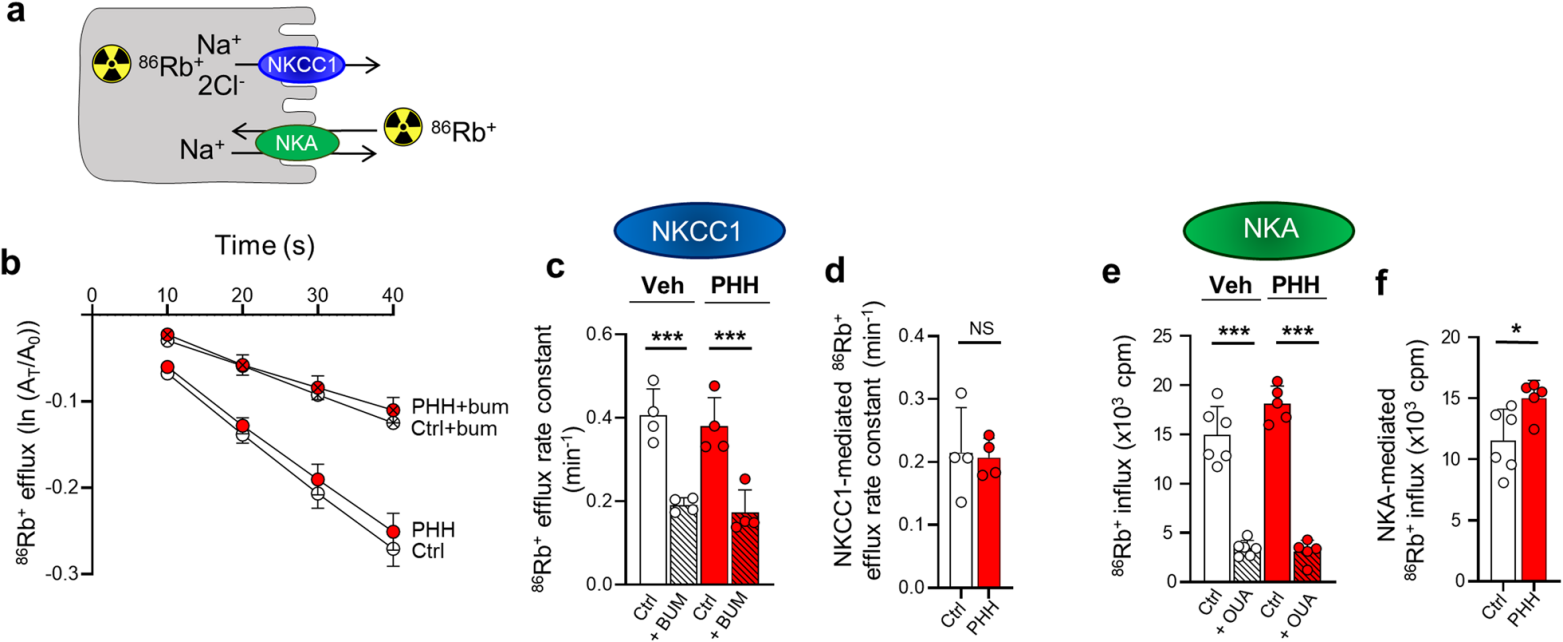
PHH associates with Na^+^/K^+^-ATPase hyperactivity. **a** Schematic illustration of the principle behind the ^86^Rb^+^ isotope flux assays. ^86^Rb^+^ is a congener for K^+^ and can be used to quantify the transport activity. **b** Loss of ^86^Rb^+^ from the choroid plexus as a function of time in control rats (n = 4) and PHH rats (n = 4) in presence or absence of 20 µM bumetanide. The y-axis is the natural logarithm of the choroidal ^86^Rb^+^ amount left at time T (A_T_) divided by the initial amount at time 0 (A_0_). **c** Efflux rates for ^86^Rb^+^ in control rats (n = 4) and PHH rats (n = 4) in presence of absence of 20 µM bumetanide. **d** NKCC1-mediated efflux rates (bumetanide-sensitive fractions) for ^86^Rb^+^ in control rats (n = 4) and PHH rats (n = 4). **e** ^86^Rb^+^ influx (measured in counts per min; cpm) in control rats (n = 6) and PHH rats (n = 5) in presence or absence of 2 mM ouabain. **f** NKA-mediated ^86^Rb^+^ influx (ouabain-sensitive fractions) in control rats (n = 6) and PHH rats (n = 5). Error bars represent standard deviation and statistical significance was tested with an unpaired two-tailed t-test or a one-way ANOVA followed by Sidak’s multiple comparisons test (**c** and **e**). *P < 0.05, ***P < 0.001, *NS* not significant

To determine PHH-mediated modulation of the choroidal transport activity of NKCC1 and the Na^+^/K^+^-ATPase, we performed isotope-based ex vivo transport assays on choroid plexus acutely isolated from PHH and control rats 24 h post-IVH. ^86^Rb^+^ acts as a congener for K^+^ and can, as such, replace the K^+^ binding in these transporters and represent a read-out of transport activity (Fig. 2a). The outward transport by NKCC1 was monitored as choroidal ^86^Rb^+^ efflux as a function of time (Fig. 2b). The ^86^Rb^+^ efflux rate was diminished by ~ 50% in presence of the NKCC1 inhibitor bumetanide in both control and PHH rats (control: 0.41 ± 0.06 min^−1^ vs. 0.19 ± 0.02 min^−1^; PHH: 0.38 ± 0.07 min^−1^ vs. 0.17 ± 0.05 min^−1^, n = 4, *P* < 0.001, Fig. 2c). The NKCC1-mediated ^86^Rb^+^ efflux rate (the bumetanide-sensitive fractions) were similar whether obtained in choroid plexus from PHH rats or their control counterparts (0.22 ± 0.07 min^−1^ in control rats vs. 0.21 ± 0.03 min^−1^ in PHH rats, n = 4, *P* = 0.84, Fig. 2d). The choroidal NKCC1-mediated transport activity thus appears unaltered in ex vivo choroid plexus obtained from the present experimental model of PHH. The transport activity of the Na^+^/K^+^-ATPase, assessed by the amount of ^86^Rb^+^ influx, was diminished by ~ 80% in presence of the Na^+^/K^+^-ATPase inhibitor ouabain, n = 5–6, *P* < 0.001 (Fig. 2e), demonstrating that the vast majority of the ^86^Rb^+^ influx occurred via the Na^+^/K^+^-ATPase. The Na^+^/K^+^-ATPase-mediated ^86^Rb^+^ influx (the ouabain-sensitive fraction) was significantly increased in PHH rats compared to control rats (15.0 ± 1.5 × 10^3^ cpm in PHH vs. 11.5 ± 2.6 × 10^3^ cpm in control, n = 5–6, *P* < 0.05, Fig. 2f). Our results demonstrate that IVH causes ex vivo choroidal hyperactivity of the Na^+^/K^+^-ATPase, which thus may contribute to the observed CSF hypersecretion.

### Blood or its component, hemin, do not affect the choroidal transporters

**Fig. 3:**
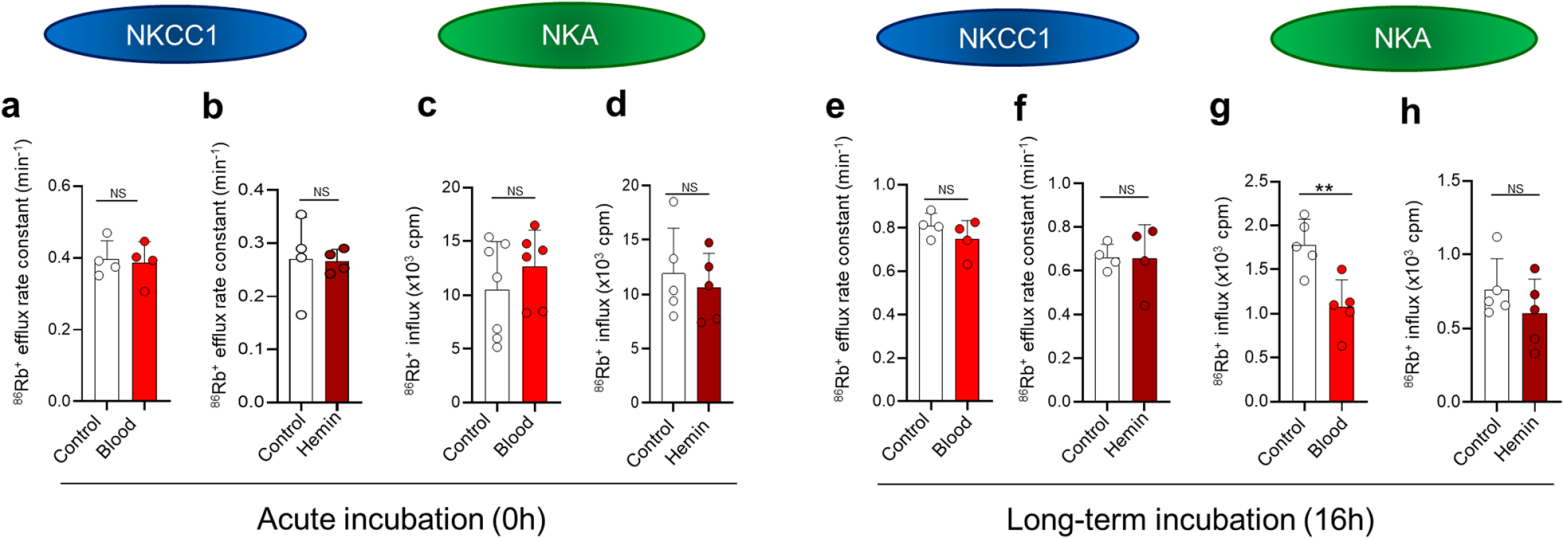
Blood and its breakdown products do not hyperactivate NKCC1 or the Na^+^/K^+^-ATPase in ex vivo choroid plexus. **a** Loss of ^86^Rb^+^ from the choroid plexus shown as efflux rate constants for ^86^Rb^+^ in control (n = 4) and after acute exposure to blood (20% of surrounding fluid, n = 4). **b** Efflux rate constants for ^86^Rb^+^ in control and after acute exposure to 50 µM hemin (n = 4 of each). **c** ^86^Rb^+^ influx in control and after acute exposure to blood (20% of surrounding fluid), n = 6–7. **d** ^86^Rb^+^ influx in control and after acute exposure to 50 µM hemin (n = 5 of each). **e** Efflux rate constants for ^86^Rb^+^ in control and after long-term exposure (16 h) to blood (20% of surrounding fluid), n = 4 of each. **f** Efflux rate constants for ^86^Rb^+^ in control and after long-term exposure (16 h) to 50 µM hemin (n = 4 of each). **g** ^86^Rb^+^ influx in control and after long-term exposure (16 h) to blood (20% of surrounding fluid), n = 5 of each. **h** ^86^Rb^+^ influx in control and after long-term exposure (16 h) to 50 µM hemin (n = 5 of each). Error bars represent standard deviation and statistical significance was tested with an unpaired two-tailed t-test. **P < 0.01, *NS* not significant

To elucidate whether the mere presence of blood in the fluid surrounding the choroid plexus could elevate transporter activity, we determined the transport rate by ^86^Rb^+^ flux assays of NKCC1 and the Na^+^/K^+^-ATPase in choroid plexus acutely isolated from naïve rats exposed to autologous blood or its break-down product hemin. Acute exposure of the isolated choroid plexus to blood did not alter the choroidal ^86^Rb^+^ efflux rate (0.40 ± 0.05 min^−1^ with aCSF control condition vs. 0.39 ± 0.06 min^−1^ with blood exposure, n = 4, *P* = 0.81, Fig. 3a) nor did exposure to hemin (0.27 ± 0.08 min^−1^ vs. 0.27 ± 0.02 min^−1^, n = 4, *P* = 0.91, Fig. 3b). As observed for NKCC1, the choroidal ^86^Rb^+^ influx was undisturbed by acute exposure to blood (12.6 ± 3.4 × 10^3^ cpm, n = 6, compared to control 10.6 ± 4.4 × 10^3^ cpm, n = 7, *P* = 0.37, Fig. 3c) or hemin (10.7 ± 3.1 × 10^3^ cpm, n = 5, compared to control 11.9 ± 4.2 × 10^3^ cpm, n = 5, *P* = 0.6, Fig. 3d). Our results indicate that the transport rate of neither NKCC1 nor the Na^+^/K^+^-ATPase is augmented by acute exposure to blood or its break-down product hemin.

### Prolonged exposure of ex vivo choroid plexus to blood does not alter the transport rate of NKCC1 or the Na^+^/K^+^-ATPase

**Fig. 4:**
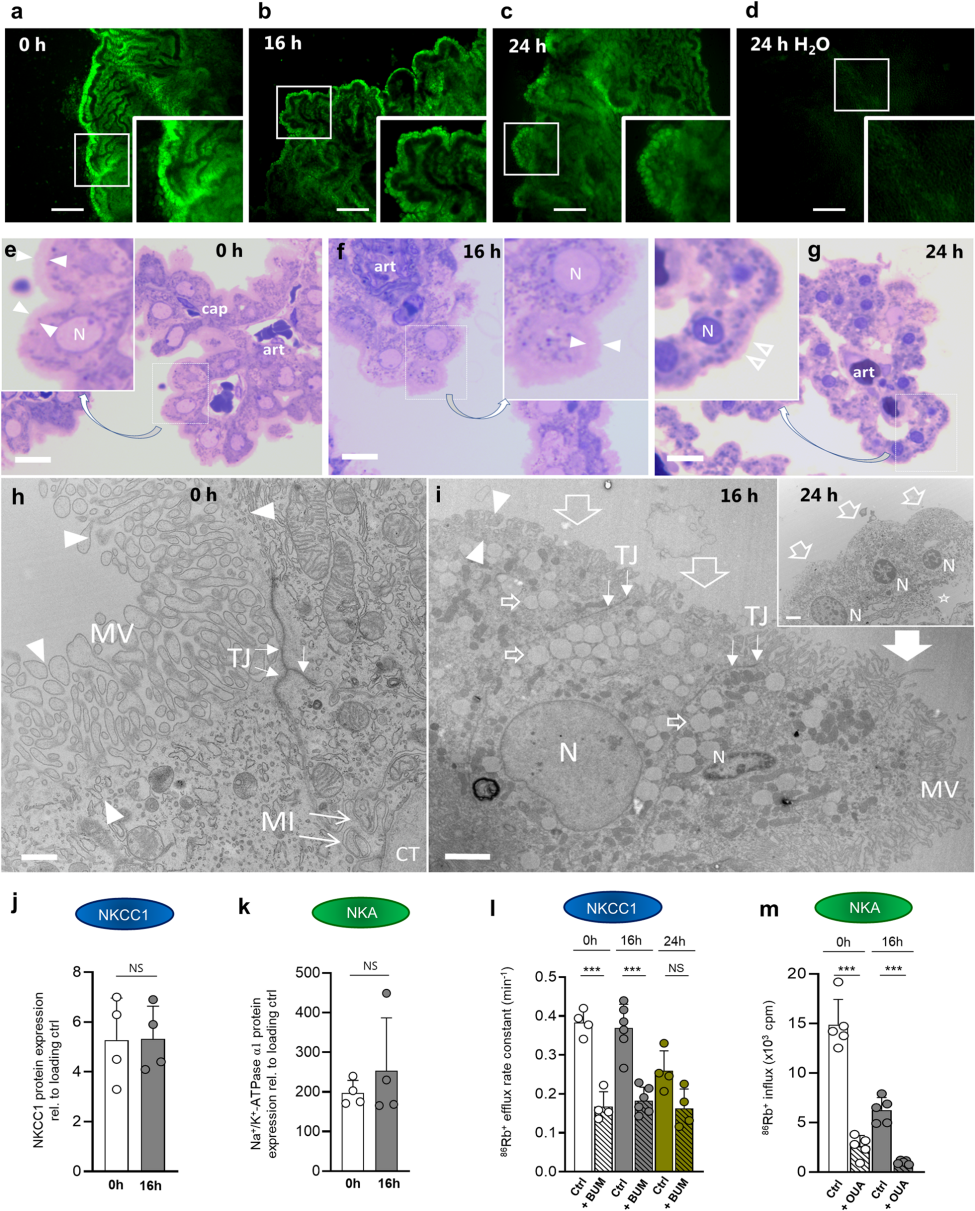
Isolated choroid plexus is viable and retains it transport activity upon culturing. Isolated rat choroid plexus demonstrated calcein fluorescence, indicative of viable cells, upon tissue culturing for 0, 16 and 24 h (**a**–**c**), whereas fluorescence was absent in control choroid plexus kept in H_2_O for 24 h (**d**). Inserts are magnification of the white boxes. Scale bars 500 μm. Choroid plexus tissue fixed directly after isolation (**e**) or following 16 h (**f**) or 24 h (**g**) of tissue culture and stained with toluidine blue. The boxed areas have been enlarged (insets) to reveal nuclear detail and presence of an organelle-free apical zone that includes the brush border (bounded by arrowheads). Note that after 24 h of culture, the brush border is greatly diminished (open arrowheads), and epithelial cells display small, pyknotic nuclei. Art, arteriole; cap, capillary; N, nucleus. Scale bars 5 mm. Electron micrographs of the choroid plexus acutely isolated (**h**) or after 16 h of tissue culture (**i**). At both time points tight junctions (TJ; small, white arrows) and basolateral membrane invaginations (MI; pointed arrows) are well developed, and the extracellular basal membrane remains juxtaposed to underlying loose connective tissue (CT). In tissue cultured for 16 h (**i**), there is a diminution or disappearance of microvilli in many cell profiles (large, open arrows), whereas other cells retain microvilli (large, closed arrow). Most nuclei are euchromatic, but chromatin condensation has commenced in a minority of cells (nucleus on the right), and in the cytoplasm there are large, empty vacuoles (small, open arrows), but organelles remain intact. Inset in **i** shows the epithelium after 24 h of culture. Although tight junctions are intact, there are no microvilli left, nuclei are pyknotic, cytolysis has commenced, basal membrane invaginations have disappeared, and the contact with underlying extracellular matrix has been lost (asterisk). Scale bars 500 nm; 2 μm; 2 μm. Quantification of Western blots of acutely isolated rat choroid plexus (0 h) and following tissue culturing (16 h) with anti-NKCC1 antibody (**j**) or the α1 subunit of the Na^+^/K^+^-ATPase (**k**). Data illustrated as normalized to GAPDH. **l** ^86^Rb^+^ efflux rates obtained in choroid plexus acutely isolated (0 h, n = 4) or after tissue culturing (16 h, n = 6; 24 h, n = 4) with or without the NKCC1 inhibitor bumetanide (BUM, 20 µM). **m** ^86^Rb^+^ influx obtained in choroid plexus acutely isolated (0 h, n = 5) or after 16 h tissue culturing with or without the Na^+^/K^+^-ATPase inhibitor ouabain (OUA, 2 mM). Error bars represent standard deviation and statistical significance was tested with an unpaired two-tailed t-test or a one-way ANOVA followed by Sidak’s multiple comparisons test (**l** and **m**). ***P < 0.001, *NS* not significant.

PHH usually develops over hours to days and blood exposure may thus exert its effect on the choroidal tissue on a similar time scale. We therefore established a culture regime for acutely isolated intact choroid plexus with which the effects of prolonged exposure to blood or its break-down product hemin could be determined. The choroid plexus is a monolayer of epithelial cells, which are all in contact with the gas-equilibrated culture medium as in conventional cell culture. The viability of the cultured choroid plexus epithelium was confirmed with a calcein-AM survival assay [33], which demonstrated live epithelium at the tested time scales 0, 16 and 24 h (Fig. 4a–c). A choroid plexus placed in sterile dH_2_O served as a negative control (Fig. 4d). To reveal the morphology of cultured choroid plexus epithelium, we performed light and transmission electron microscopy of acutely isolated choroid plexus in comparison to those cultured for 16 or 24 h. After 16 h of tissue culture, the epithelial cells retained their tight junctional coupling and basolateral invaginations. The extracellular basal membrane remained juxtaposed to underlying loose connective tissue, the organelles were intact, and most nuclei were euchromatic (Fig. 4e, f, h, i). However, some cells displayed empty cytosolic vacuoles and diminution of microvilli (Fig. 4f, i). After 24 h tissue culturing, the choroid plexus epithelial cells retained their tight junctions, but had lost microvilli and basal membrane invaginations and displayed small, pyknotic nuclei and signs of initial cytolysis (Fig. 4g, i inset). These data demonstrate that the cultured choroid plexus retains its overall structural components, such as intact nuclei, brush border, and general barrier function with 16 h tissue culturing, whereas these differentiated features disappear with longer incubation times. Prior to commencing ^86^Rb^+^ flux assays, the continued presence of the transporters NKCC1 and the Na^+^/K^+^-ATPase was verified by Western blotting demonstrating comparable transporter abundance after 16 h of culturing, n = 4, *P* = 1.0 and *P* = 0.4 respectively (Fig. 4j, k and Additional file 1, panel b). Application of the NKCC1 inhibitor bumetanide significantly (*P* < 0.001) decreased the ^86^Rb^+^ efflux rate at time points t = 0 h and t = 16 h (0 h: 0.38 ± 0.03 min^−1^ vs. 0.17 ± 0.04 min^−1^, n = 4; 16 h: 0.37 ± 0.06 min^−1^ vs. 0.18 ± 0.03 min^−1^, n = 6) and the bumetanide-sensitive ^86^Rb^+^ efflux (the NKCC1 activity) was thus similar (P = 0.49), Fig. 4l. However, at t = 24 h, the bumetanide inhibition was no longer significant (*P* = 0.11) and most NKCC1-mediated ^86^Rb^+^ efflux was thus abolished upon lengthy culturing. Ouabain-sensitive ^86^Rb^+^ influx (the Na^+^/K^+^-ATPase activity) persisted after 16 h tissue culturing of the choroid plexus (Fig. 4m), although with lesser activity (0 h: 12.3 ± 1.8 × 10^3^ cpm vs. 16 h: 5.2 ± 1.1 × 10^3^ cpm, n = 5 of each, *P* < 0.001). These data suggest that the choroid plexus is viable with persistent transport mechanisms after 16 h in culture medium and can be employed to elucidate effects on prolonged exposure to blood or its break-down product hemin.

NKCC1 activity assessed by ^86^Rb^+^ efflux was not significantly affected by 16 h exposure to blood (0.75 ± 0.09 min^−1^ vs. 0.81 ± 0.06 min^−1^ in control, n = 4, *P* = 0.27, Fig. 3e) nor hemin (0.66 ± 0.15 min^−1^ vs. 0.66 ± 0.06 min^−1^ in control, n = 4, *P* = 0.95, Fig. 3f). Na^+^/K^+^-ATPase activity assessed by ^86^Rb^+^ influx was not elevated upon 16 h exposure of the choroid plexus to blood, rather; it was reduced (1.08 ± 0.31 × 10^3^ cpm vs. 1.78 ± 0.29 × 10^3^ cpm in control, n = 5, *P* < 0.01, Fig. 3g), whereas hemin exposure did not yield any significant changes in ^86^Rb^+^ uptake (0.61 ± 0.23 × 10^3^ cpm vs. 0.77 ± 0.24 × 10^3^ cpm in control, n = 5, *P* = 0.28, Fig. 3h). Taken together, our results suggest that blood, or its break-down product hemin, do not in themselves induce transporter hyperactivity in ex vivo choroid plexus.

### Elevated inflammation in CSF from PHH patients

**Fig. 5:**
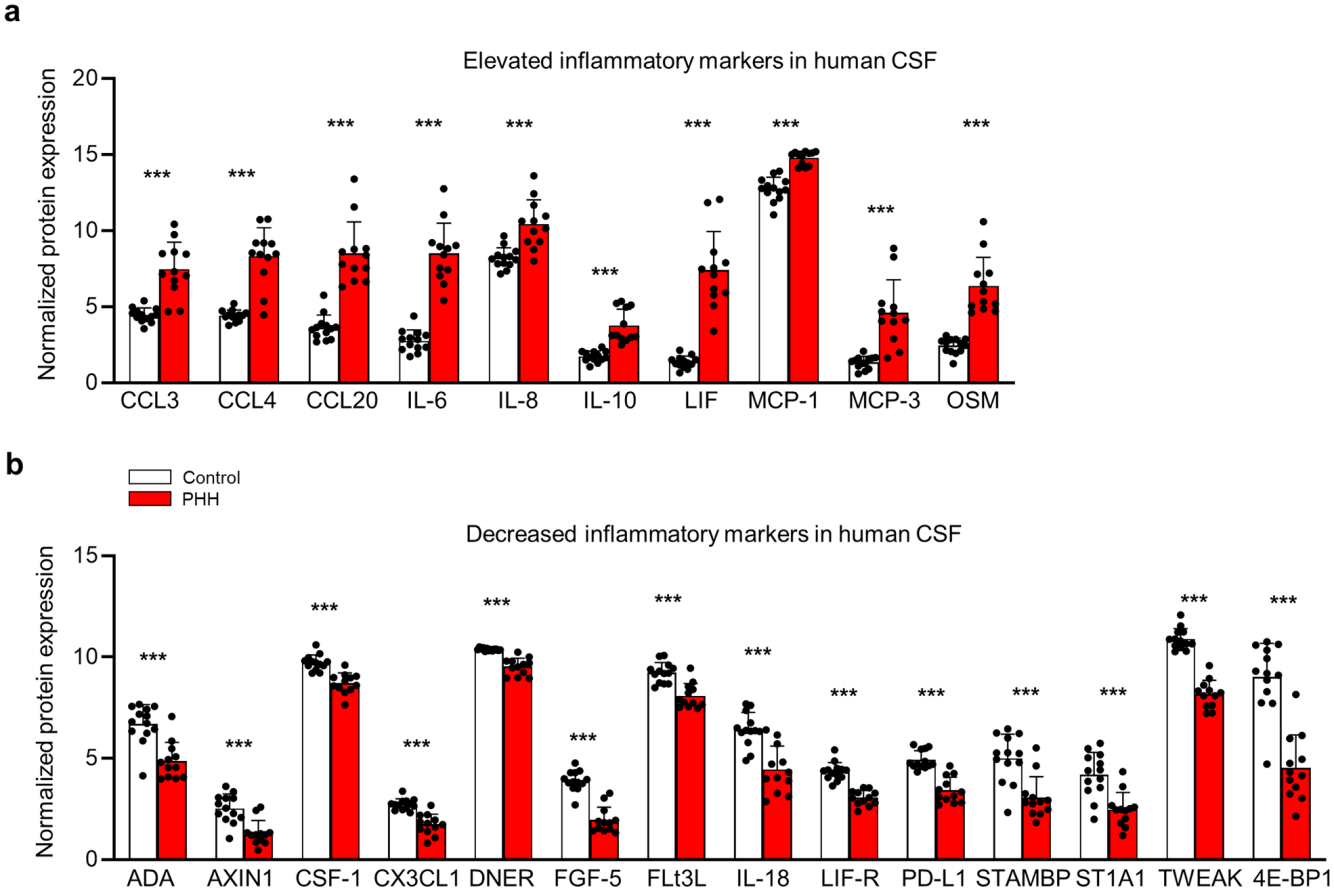
Elevated inflammation in CSF from PHH patients. **a** 10 inflammatory markers were elevated in CSF from PHH patients (n = 12) compared to healthy control subjects (n = 13). **b** 14 inflammatory markers were decreased in PHH patients compared to healthy control subjects. Data are presented as normalized protein expression (NPX) values (mean ± SD) and analyzed with an unpaired two-tailed t-test or a Mann-Whitney test with Bonferroni correction applied to accommodate multiple comparisons. ***P < 0.001

The IVH-mediated CSF hypersecretion observed in the rodent PHH model, accordingly, appears to not originate directly from the blood components acting on the choroid plexus. However, such pathophysiology could arise from an inflammatory response that develops secondarily to the hemorrhagic event. To characterize the inflammatory response associated with PHH, we quantified inflammatory markers in CSF from PHH rats (n = 17) and control rats (n = 17) collected 24 h post IVH using the proximity extension assay (PEA) technique, which relies on dual antibody-based recognition for detection of 92 different inflammatory markers [34]. However, the vast majority of the inflammatory markers were simply undetectable in the rat CSF samples and only one inflammatory marker, monocyte chemotactic protein 4 (MCP-4), was significantly elevated in the CSF from PHH rats compared to control rats (PHH: 3.35 ± 0.65 normalized protein expression (NPX) versus control: 1.51 ± 0.40 NPX, n = 17, *P* < 0.001, Additional file 4). As the PEA technique employs antibodies that recognize the human version of the inflammatory markers, we quantified the inflammatory marker content in CSF from 12 human PHH patients and 13 healthy control subjects (see "Methods" section for clinical characteristics of the experimental groups), Additional file 5. The CSF levels of 10 inflammatory markers were significantly elevated in PHH patients compared to healthy control subjects: CCL3, CCL4, CCL20, IL-6, IL-10, leukemia inhibitory factor (LIF), MCP-1, OSM, MCP-3, and IL-8 (Fig. 5a and Additional file 6). The CSF levels of 14 inflammatory markers were significantly decreased in the CSF from PHH patients compared to healthy control subjects: fractalkine (CX3CL1), delta and notch-like epidermal growth factor-related receptor (DNER), fibroblast growth factor 5 (FGF-5), fms-related tyrosine kinase 3 ligand (Flt3L), IL-18, LIF-R, programmed cell death ligand 1 (PD-L1), tumor necrosis factor ligand superfamily member 12 (TWEAK), eukaryotic translation initiation factor 4E-binding protein 1 (4E-BP1), macrophage colony-stimulating factor 1 (CSF-1), adenosine deaminase (ADA), sulfotransferase 1A1 (ST1A1), axin-1 (AXIN1), and STAM-binding protein (STAMBP) (Fig. 5b and Additional file 7). Taken together, these findings demonstrate that PHH is associated with an altered CSF composition of inflammatory markers indicating that hemorrhage promotes an inflammatory response within the brain ventricles that may act on the choroid plexus.

### Upregulation of the choroid plexus immune machinery in PHH rats

**Fig. 6:**
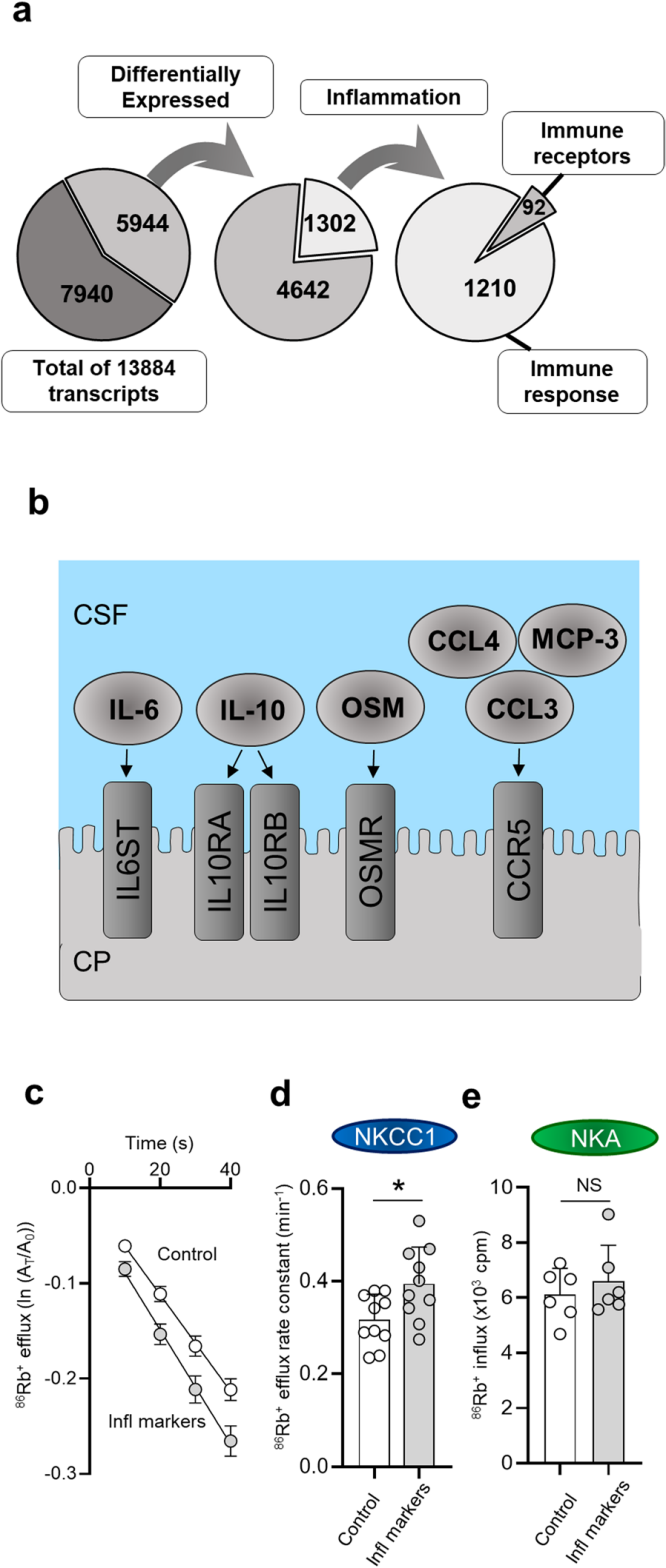
Upregulation of the choroidal immune machinery in PHH rats and NKCC1 hyperactivity. **a** RNAseq of choroid plexus from PHH and control rats revealed transcription of 13,884 genes. Of the transcripts detected at ≥ 0.5 TPM, 5944 were considered differentially transcribed. A total of 1302 transcripts were linked to inflammation, 92 encoded immune receptors, while the remaining 1210 encoded other inflammatory agents involved in mediating immune responses. **b** Schematic illustration of those inflammatory markers found elevated in the CSF from PHH patients that also displayed upregulated transcription of their corresponding receptors (or co-receptors) in the rat choroid plexus after IVH. **c** Choroidal ^86^Rb^+^ efflux after 16 h incubation in absence (control, n = 5) or presence (n = 5) of a mix of inflammatory markers (CCL3, OSM, IL-10 and IL-6, all at 500 ng/ml), with the efflux rate constants plotted in **d**, **e** Choroidal ^86^Rb^+^ influx after 16 h incubation in absence (control, n = 5) or presence (n = 5) of a mix of inflammatory markers (CCL3, OSM, IL-10 and IL-6, all at 500 ng/ml). Error bars represent standard deviation and statistical significance was tested with an unpaired two-tailed t-test. *P < 0.05, *NS* not significant

To determine whether PHH, possibly via its associated inflammatory response, altered the rat choroid plexus transcriptome, and thus potentially its CSF secretory properties, we searched the 5944 differentially transcribed genes (described above, Additional file 3) using pre-determined key words (see "Methods" section) to identify transcripts encoding proteins with functions directly or indirectly linked to inflammation. A total of 1302 transcripts were linked to inflammation, 98% of which were upregulated in PHH versus control. 92 of these transcripts encoded immune receptors (Additional file 8), while the remaining 1,210 transcripts encoded other inflammatory agents involved in mediating immune responses (Additional file 9 and Fig. 6a). Among the 1,210 transcripts encoding other inflammatory agents involved in mediating immune responses were subunits of the NF-κB transcription factor complex (NFKB1, NFKB2, RELA, RELB, Additional file 9), indicating activation of the inflammatory NF-κB signaling pathway. Among the upregulated immune receptors were those activated by (or acting as co-receptors for) IL-6 (IL6ST), IL-10 (IL10RA, IL10RB), and OSM (OSMR), all of which were elevated in the CSF from PHH patients, as well as C-C motif chemokine receptor 5 (CCR5), capable of binding the elevated CSF inflammatory markers CCL3, CCL4, and MCP-3 (Figs. 5a and 6b). Taken together, our findings indicate that PHH is associated with inflammatory alterations of the choroidal transcriptome.

### PHH-induced inflammatory markers promote choroidal NKCC1 hyperactivity ex vivo

To resolve if the PHH-modulated inflammatory pathways could cause hyperactivation of the choroidal transporters, we exposed acutely isolated choroid plexus to the set of inflammatory markers (in the rat version) that were both elevated in the SAH patient CSF and also target the inflammatory receptors that were upregulated in choroid plexus from IVH rats (Fig. 6b). The mix of inflammatory markers comprised CCL3, OSM, IL-10 and IL-6 (500 ng/ml of each), which in combination are predicted to activate the receptors depicted in Fig. 6b. After 16 h incubation, the transport activity of NKCC1 and the Na^+^/K^+^-ATPase was determined with ^86^Rb^+^ flux assays. The ^86^Rb^+^ efflux comprising the NKCC1 activity was increased upon 16 h exposure to this mix of inflammatory markers (0.39 ± 0.08 min^−1^ vs. 0.32 ± 0.06 min^−1^ in control, n = 10 of each, *P* < 0.05, Fig. 6c, d) in contrast to the ^86^Rb^+^ influx comprising the Na^+^/K^+^-ATPase activity (6.60 ± 1.30 × 10^3^ cpm vs. 6.12 ± 0.95 × 10^3^ cpm in control, n = 6, *P* = 0.48, Fig. 6e), which remained unaltered. In the isolated ex vivo experimental setting, the inflammatory pathways activated in PHH thus appeared to directly hyperactivate NKCC1.

## Discussion

We here demonstrate that an animal model of IVH presents with the ventriculomegaly and excessive brain water accumulation characteristic of the PHH observed in patients with cerebral hemorrhagic events [1]. We reveal that the PHH was associated with elevated CSF secretion, at least in part due to elevated activity of choroid plexus membrane transporters involved in CSF secretion.

PHH arises following hemorrhagic events of different kinds, e.g. SAH and IVH [1]. The condition has generally been assumed to occur following blockage of the CSF flow pathways, although a precise documentation of blockage often remains undetected on brain images collected during the diagnostic work-up of this patient group [1]. Various molecules related to hemolysis and clot formation such as hemoglobin, iron, hemin, thrombin, and peroxiredoxin 2 have been implicated in PHH development, in addition to reactive oxygen species in the preterm infants [40–45] and may contribute to depression of CSF drainage leading to undetectable micro-blockage or scarring of the CSF drainage pathways aided by the subsequent inflammatory response [9]. Alternatively, as here observed, the early phase of PHH may, in part, arise subsequent to IVH-induced hypersecretion of CSF [9, 14], as also observed with ischemic stroke-induced hyperactivation of NKCC1 in choroid plexus [46] and aligned with inhibition of NKCC1 ameliorating IVH-mediated ventriculomegaly in rats [14, 47]. These findings align with the CSF hypersecretion leading to the ventriculomegaly observed in patients with choroidal hyperplasia and choroid plexus papilloma [17, 18], and can be simulated by experimental mimicry of CSF hypersecretion in rats [14, 48]. The observed IVH-induced increase in brain fluid content and the associated ventriculomegaly were not reflected in a significant ICP elevation in the experimental rats, although a tendency was evident. With the cranial compliance, the short time span of 24 h between the experimental hemorrhagic event and the ICP measurements may not suffice for adequate fluid accumulation to robustly elevate ICP in the rat model or, alternatively, the ICP may already have been significantly elevated at an earlier time point and is trending downwards at 24 h post IVH.

CSF secretion by the choroid plexus occurs, in part, by the action of NKCC1 and the Na^+^/K^+^-ATPase [14, 15, 48–51], both localized to the luminal membrane of choroid plexus facing the CSF [52]. These were elevated at the transcriptional level in choroid plexus excised from the IVH rats and their transport activity may, in addition, be modulated by post-translational modifications, e.g., phosphorylation. Such phosphorylation-dependent upregulation of NKCC1-mediated CSF secretion was demonstrated to underlie the IVH-induced CSF hypersecretion in an earlier in vivo model of PHH [14]. In our ex vivo conditions, Na^+^/K^+^-ATPase activity was elevated in choroid plexus acutely excised from PHH rats. Such difference could be explained by the different experimental approaches, i.e., in vivo determination of NKCC1-mediated CSF secretion [14] versus isotope-based transporter assays in acutely excised choroid plexus obtained from these animals 24 h after IVH, in which components present in the CSF in vivo may be absent. It should be noted that increasing Na^+^/K^+^-ATPase activity may mask the effect of PHH-induced increased NKCC1 activity by lowering intracellular Na^+^, reducing the electrochemical gradient for efflux via the co-transporter. Elevated Na^+^/K^+^-ATPase activity in the choroid plexus from IVH rats could arise by a similar phosphorylation-based increase in activity [53], by the observed elevated transcription of the α1 isoform of the Na^+^/K^+^-ATPase, which is highly expressed in the choroid plexus [48, 54], or by emergence of the Na^+^/K^+^-ATPase α2 isoform, which is expressed at substantially lower levels than the α1 isoform [48], see Additional file 3. Our RNAseq data demonstrated an IVH-mediated 46% increase in α2 transcript abundance (*ATP1A2*, Additional file 3). This Na^+^/K^+^-ATPase isoform has previously been implicated in LPS/TLR4/NF-κB-mediated immune responses [55, 56], a form of which represents a key step in the previous animal model of IVH-induced, NKCC1-mediated ventriculomegaly [14]. The IVH-induced CSF hypersecretion may thus arise following complex and potentially complementary signaling pathways and transport mechanisms that could well involve additional choroidal transporters, e.g. NCBE, which was here found to be upregulated at the transcriptional level in alignment with an earlier study [57].

We demonstrated that the IVH-mediated CSF hypersecretion mostly likely does not arise as a direct function of choroidal exposure to blood, or its breakdown product hemin [an established TLR4 agonist [58], as these failed to activate NKCC1 or the Na^+^/K^+^-ATPase when applied to choroid plexus ex vivo. Choroidal hypersecretion could, instead, arise as a function of the inflammatory response associated with the hemorrhagic event. Morphological examination of choroid plexus revealed that these retained their structural integrity following the hemorrhagic event but displayed transcriptional upregulation of a wealth of inflammatory markers and immune receptors, including different subunits of the NF-κB transcription factor complex, which has previously been implicated in IVH-mediated CSF hypersecretion [14]. Our findings thus corroborate the notion that hemorrhage promotes an inflammatory response in the choroid plexus, which could either originate in the choroid plexus upon its interaction with the ventricular blood or by inflammatory markers present in the CSF as a direct consequence of the hemorrhagic event.

We here performed an unbiased screening of 92 different inflammatory markers in ventricular samples of CSF obtained during the diagnostic workup of a set of SAH patients with later development of PHH. These CSF samples were compared to CSF collected from healthy control individuals undergoing prophylactic neurosurgical removal of unruptured aneurysms. As it is considered unethical to access the ventricular compartment of healthy individuals without a clinical indication, the CSF from this control group was collected from the basal cisterns during the neurosurgical procedure. The different CSF collection sites in the two test groups is ethically unavoidable but must be considered a limitation of the present study. The CSF obtained from the SAH patients presented both up- and downregulation of various inflammatory markers. The majority of the upregulated inflammatory markers were chemokines (CCL3, CCL4, CCL20, MCP-1, and MCP-3) or interleukins (IL-6, IL-8, and IL-10), which promote and regulate immune responses in a pleiotropic manner, depending on their target [59, 60]. The upregulated inflammatory marker IL-6 has been implicated in various neuroinflammatory disorders [61] and elevated IL-6 levels correlate with SAH-related pathological brain changes such as delayed cerebral ischemia, cerebral vasospasm, and infections [62, 63]. A recent literature review reported IL-6 as one of the inflammatory markers associated with most evidence of upregulation in PHH patients [64] and IL-6 has been proposed as a prognostic marker for chronic hydrocephalus development following SAH [65]. The upregulated chemokine CCL3 has likewise been reported elevated in CSF from PHH patients [66] and its expression increases markedly at sites of tissue injury where it, among other things, chemoattracts different immune cells [67, 68]. CCL3 may thus potentially contribute to the inflammatory changes observed in the PHH brain. Some of the other upregulated inflammatory markers, IL-8 and MCP-1, which both chemoattract immune cells to sites of inflammation [60, 69], have, like IL-6 and CCL3, been reported elevated in CSF from PHH patients [70]. Whether these play a role in the pathogenesis of PHH remains unresolved, as others report unaltered levels [66]. Interestingly, IL-18, an inflammatory marker previously demonstrated to be elevated in CSF from PHH patients [64] was found downregulated in the present study. This discrepancy may arise from differences in analytical methods (ELISA in the previous studies [71, 72] and PEA in the present study) or origin of the analyzed CSF samples (ventricular vs. lumbar in the previous studies [71, 72] and ventricular vs. cisternal in the present), and the role of IL-18 in PHH therefore needs further elucidation. The downregulated inflammatory markers were heterogeneous in nature compared to the upregulated inflammatory markers, comprising cytokines, growth factors, enzymes, and other proteins directly or indirectly linked to inflammation [60, 73–78].

Direct exposure of isolated rat choroid plexus to the rat version of the inflammatory markers observed in SAH patient CSF, the receptors of which were also upregulated at the transcriptional level in IVH rats, led to NKCC1 hyperactivity ex vivo. It should be noted that these results were obtained with only a subset of the 10 elevated inflammatory markers acting on only a subset of the 92 immune receptors with increased transcript abundance in the rat choroid plexus. We selected those that were upregulated in both CSF and choroid plexus to obtain a proof of concept. The remaining elevated inflammatory markers could well promote a similar NKCC1 activation even without transcriptional upregulation of their receptors and vice versa, alone or in combination. It thus appears that a hemorrhage-induced inflammatory response enables the transporter hyperactivation leading to the CSF hypersecretion observed in connection with the hemorrhagic event [14]. Its complexity appears to be, at least partially, recapitulated in the ex vivo conditions. We hypothesize that choroid plexus requires prolonged exposure to these inflammatory markers for these to serve their purpose. To that end, we established a tissue culture regime, in which choroid plexus retained its morphology, structural integrity, and transport activity for 16 h and thereby allowed for prolonged exposure to modulatory factors. At 24 h, the cellular integrity of the choroid plexus epithelial cells was no longer comparable to the in vivo choroid plexus. One may consider that choroid plexus epithelial cells kept as primary cell culture or as immortalized cell lines [79–81] may lack some of the characteristics of choroid plexus epithelial cells, which may affect physiological aspects and protein expression of the choroid plexus epithelial cells [79].

Secretion and circulation of CSF in quadrupeds may differ from that of human. Therefore, limitations to the study include the use of rodents for the invasive in vivo quantification of CSF secretion rates and for all ex vivo determinations of choroid plexus transcript abundance and transport activity. These processes cannot be studied in the humans. An additional limitation is that the human patient group consists of aged individuals (limited by those that were admitted to the hospital with SAH), whereas the basic mechanisms of IVH-induced PHH formation were performed on young adult rats (9 weeks). We cannot rule out that age influences the molecular mechanisms underlying IVH-induced PHH and that the contribution of drainage blocking versus CSF hypersecretion may vary over time after the hemorrhagic event and with the type event (SAH, IVH or intracranial hemorrhage).

In conclusion, a component of PHH development may be linked to CSF hypersecretion, due, in part, to choroid plexus Na^+^/K^+^-ATPase and NKCC1 hyperactivation. The underlying molecular coupling remains unresolved but is likely to be associated with the inflammatory response observed with a hemorrhagic event in patient CSF and rat choroid plexus. Hemorrhage-mediated hypersecretion may serve to swiftly rid the ventricular system of the blood introduced with the hemorrhage, but in some cases promote the ventriculomegaly and elevated ICP observed in this patient group. Future revelation of the molecular etiology underlying PHH-mediated modulation of CSF secretion and the associated regulatory immune pathways may provide rational therapeutic targets towards pharmacological treatment of this condition.

## Supplementary Information

**Additional** **file** **1:** **Figure** **S1.** Fluxassay, Western blot.

**Additional** **file** **2:** **Figure** **S2.** Flowdiagram for the CSF analysis. 

**Additional** **file** **3:** **Table** **S1.** RNAseq_PHH_rat_all genes.

**Additional** **file** **4:** **Table** **S2.** Inflammatory markers for statistical analysis in rat CSF samples.

**Additional** **file** **5:** **Table** **S3.** Inflammatory markers for statistical analysis in human CSF samples.

**Additional** **file** **6:** **Table** **S4.** Elevated inflammatory markers in CSF from PHH patients versus healthy control subjects.

**Additional** **file** **7:** **Table** **S5.** Decreased inflammatory markers in CSF from PHH patients versus healthy control subjects.

**Additional** **file** **8:** **Table** **S6.** RNAseq_rats_PHH_Immune_Receptors.

**Additional** **file** **9:** **Table** **S7.** RNAseq_rats PHH_Immune_Response.

## Data Availability

The datasets used and/or analyzed during the current study are available from the corresponding author on reasonable request.

## Abbreviations

aCSF: Artificial CSF
ADA: Adenosine deaminase
AQP1: Aquaporin 1
AXIN1: Axin-1
CCL: C-C motif chemokine ligand
CCR: C-C motif chemokine receptor
CSF: Cerebrospinal fluid
CSF-1: Macrophage colony-stimulating factor 1
CX3CL1: Fractalkine
DNER: Delta and notch-like epidermal growth factor-related receptor
EVD: External ventricular drain
FGF: Fibroblast growth factor
FLt3L: Fms-related tyrosine kinase 3 ligand
ICP: Intracranial pressure
IL: Interleukin
IVH: Intraventricular hemorrhage
LIF: Leukemia inhibitory factor
LOD: Limit of detection
LPS: Lipopolysaccharide
MCP: Monocyte chemotactic protein
MRI: Magnetic resonance imaging
NCBE: Na^+^-coupled bicarbonate exchanger
NF-κB: Nuclear factor-κB
NKA: Na^+^/K^+^-ATPase
NKCC1: Na^+^/K^+^/2Cl^−^ cotransporter
NPX: Normalized protein expression
OSM: Oncostatin-M
PD-L1: Programmed cell death ligand 1
PEA: Proximity extension assay
PHH: Posthemorrhagic hydrocephalus
RNAseq: RNA sequencing
SAH: Subarachnoid hemorrhage
STAMBP: STAM-binding protein
ST1A1: Sulfotransferase 1A1
TEM: Transmission electron microscopy
TLR4: Toll-like receptor 4
TPM: Transcripts per million
TRPV4: Transient receptor potential vanilloid 4
TWEAK: Tumor necrosis factor ligand superfamily member 12
4E-BP1: Eukaryotic translation initiation factor 4E-binding protein 1
