# Supplementary material for: Posthemorrhagic hydrocephalus associates with elevated inflammation and CSF hypersecretion via activation of choroidal transporters
Source: Fluids Barriers CNS. 2022 Aug 10;19:62. doi: 10.1186/s12987-022-00360-w (PMC9367104; doi:10.1186/s12987-022-00360-w)
Supplement: Supplementary file 1 — Additional file 1: Figure S1. Fluxassay, Western blot. [file 12987_2022_360_MOESM1_ESM.pdf]

Additional file 1

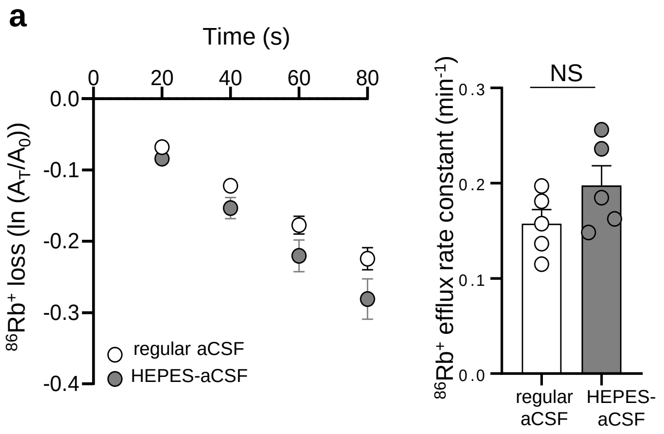

a. $^{86}Rb^{+}$ efflux from *ex vivo* rat choroid plexus conducted in regular aCSF versus HEPES-buffered aCSF (see Methods). The efflux rate constants (0.16 ± 0.03 min^{-1} in aCSF versus 0.20 ± 0.05 min^{-1} in HEPES-aCSF, n = 5 of each) were not statistically different, P = 0.16, Student's t-test. NS; not significant.

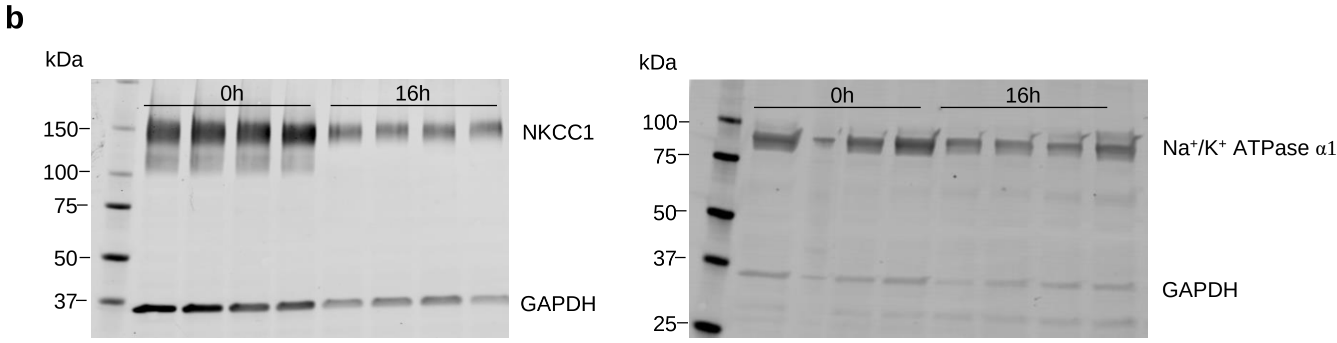

b. Western blot of choroid plexus acutely isolated or after 16h tissue culturing, stained with anti-NKCC1 or anti-Na^{+}/K^{+}-ATPase antibodies with anti-GAPDH as loading control, n = 4.
