# Supplementary figures and images for: Posthemorrhagic hydrocephalus associates with elevated inflammation and CSF hypersecretion via activation of choroidal transporters
Source: Fluids Barriers CNS. 2022 Aug 10;19:62. doi: 10.1186/s12987-022-00360-w (PMC9367104; doi:10.1186/s12987-022-00360-w)

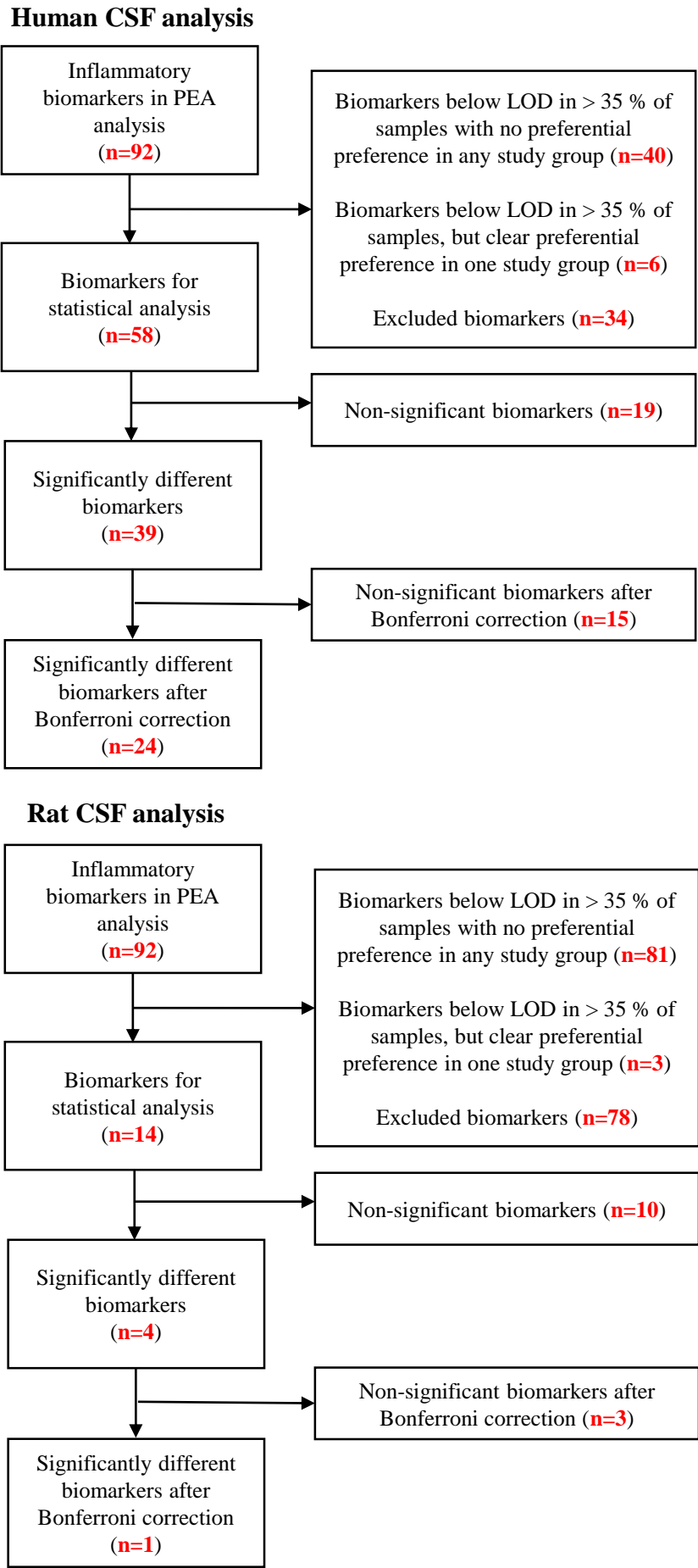

Supplement: Supplementary file 2 — Additional file 2: Figure S2. Flowdiagram for the CSF analysis. [file 12987_2022_360_MOESM2_ESM.pdf]
