# Supplementary material for: Posthemorrhagic hydrocephalus associates with elevated inflammation and CSF hypersecretion via activation of choroidal transporters
Source: Fluids Barriers CNS. 2022 Aug 10;19:62. doi: 10.1186/s12987-022-00360-w (PMC9367104; doi:10.1186/s12987-022-00360-w)
Supplement: Supplementary file 4 — Additional file 4: Table S2. Inflammatory markers for statistical analysis in rat CSF samples. [file 12987_2022_360_MOESM4_ESM.pdf]

Additional file 4. Inflammatory markers for statistical analysis in PHH rat CSF samples.

**Marker**	**Full Name**	**Statistical test**	***P* value**
MCP-4	Monocyte chemotactic protein 4	Mann-Whitney	**<0.0001**
GDNF	Glial cell line-derived neurotrophic factor	t-test (Welch's)	0.02
MCP-1	Monocyte chemotactic protein 1	t-test	0.02
DNER	Delta and Notch-like epidermal growth factor-related receptor	t-test (Welch's)	0.04
LAP TGF-beta-1	Latency-associated peptide transforming growth factor beta-1	Mann-Whitney	0.07
TWEAK	Tumor necrosis factor ligand superfamily, member 12	t-test	0.26
CXCL5	C-X-C motif chemokine 5	Mann-Whitney	0.34
OPG	Osteoprotegerin	t-test	0.38
4E-BP1	Eukaryotic translation initiation factor 4E-binding protein 1	t-test	0.66
TGF-alpha	Transforming growth factor alpha	t-test	0.70
STAMBP	STAM-binding protein	Mann-Whitney	0.71
IL-1 alpha	Interleukin-1 alpha	Mann-Whitney	0.79
FGF-21	Fibroblast growth factor 21	t-test	0.84
ARTN	Artemin	Mann-Whitney	0.89

Statistical analysis of inflammatory markers. P-values in **bold** indicate statistical significance after Bonferroni correction (0.05/14=0.004)
