# Supplementary material for: Posthemorrhagic hydrocephalus associates with elevated inflammation and CSF hypersecretion via activation of choroidal transporters
Source: Fluids Barriers CNS. 2022 Aug 10;19:62. doi: 10.1186/s12987-022-00360-w (PMC9367104; doi:10.1186/s12987-022-00360-w)
Supplement: Supplementary file 5 — Additional file 5: Table S3. Inflammatory markers for statistical analysis in human CSF samples. [file 12987_2022_360_MOESM5_ESM.pdf]

Additional file 5. Inflammatory markers for statistical analysis in human CSF samples.

**Marker**	**Full Name**	**Statistical test**	***P* value**
CCL3	C-C motif chemokine 3	t-test (Welch's)	**<0.0001**
CCL4	C-C motif chemokine 4	t-test (Welch's)	**<0.0001**
CCL20	C-C motif chemokine 20	Mann-Whitney	**<0.0001**
CSF-1	Macrophage colony-stimulating factor 1	t-test	**<0.0001**
CX3CL1	Fractalkine	t-test (Welch's)	**<0.0001**
DNER	Delta and Notch-like epidermal growth factor-related receptor	t-test (Welch's)	**<0.0001**
FGF-5	Fibroblast growth factor 5	Mann-Whitney	**<0.0001**
Flt3L	Fms-related tyrosine kinase 3 ligand	t-test	**<0.0001**
IL-6	Interleukin-6	t-test (Welch's)	**<0.0001**
IL-10	Interleukin-10	Mann-Whitney	**<0.0001**
IL-18	Interleukin-18	t-test	**<0.0001**
LIF	Leukemia inhibitory factor	t-test (Welch's)	**<0.0001**
LIF-R	Leukemia inhibitory factor receptor	t-test	**<0.0001**
MCP-1	Monocyte chemotactic protein 1	Mann-Whitney	**<0.0001**
OSM	Oncostatin-M	Mann-Whitney	**<0.0001**
PD-L1	Programmed cell death 1 ligand 1	Mann-Whitney	**<0.0001**
TWEAK	Tumor necrosis factor ligand superfamily, member 12	t-test	**<0.0001**
4E-BP1	Eukaryotic translation initiation factor 4E-binding protein 1	t-test	**<0.0001**
ADA	Adenosine Deaminase	Mann-Whitney	**<0.001**
ST1A1	Sulfotransferase 1A1	t-test	**<0.001**
MCP-3	Monocyte chemotactic protein 3	t-test (Welch's)	**<0.001**
IL-8	Interleukin-8	t-test (Welch's)	**<0.001**
AXIN1	Axin-1	Mann-Whitney	**<0.001**
STAMBP	STAM-binding protein	Mann-Whitney	**<0.001**
CD40	CD40L receptor	t-test	<0.01
TNF	Tumor necrosis factor	t-test (Welch's)	<0.01
CST5	Cystatin D	t-test (Welch's)	<0.01
IL-10RB	Interleukin-10 receptor subunit beta	t-test	<0.01
TNFRSF9	Tumor necrosis factor receptor superfamily member 9	Mann-Whitney	<0.01
CCL19	C-C motif chemokine 19	t-test	<0.01
CD5	T-cell surface glycoprotein CD5	Mann-Whitney	<0.01
CD8A	T-cell surface glycoprotein CD8 alpha chain	Mann-Whitney	<0.01
MCP-2	Monocyte chemotactic protein 2	t-test (Welch's)	<0.01
HGF	Hepatocyte growth factor	Mann-Whitney	<0.05
CXCL9	C-X-C motif chemokine 9	t-test	<0.05
CASP-8	Caspase-8	Mann-Whitney	<0.05
MMP-10	Matrix metalloproteinase-10	t-test (Welch's)	<0.05
SCF	Stem cell factor	t-test	<0.05
FGF-19	Fibroblast growth factor 19	t-test	<0.05
VEGF-A	Vascular endothelial growth factor A	t-test (Welch's)	0.07
CCL11	Eotaxin	t-test	0.08
CCL23	C-C motif chemokine 23	t-test	0.10
MMP-1	Matrix metalloproteinase-1	t-test	0.11
CXCL5	C-X-C motif chemokine 5	t-test (Welch's)	0.12
IL-18R1	Interleukin-18 receptor 1	Mann-Whitney	0.12
CXCL10	C-X-C motif chemokine 10	Mann-Whitney	0.14
CXCL6	C-X-C motif chemokine 6	t-test	0.20
EN-RAGE	Protein S100-A12	t-test	0.21
IL-12B	Interleukin-12 subunit beta	t-test	0.27
MCP-4	Monocyte chemotactic protein 4	t-test	0.28
uPA	Urokinase-type plasminogen activator	t-test	0.36
TGF-alpha	Transforming growth factor alpha	t-test (Welch's)	0.48
OPG	Osteoprotegerin	t-test (Welch's)	0.56
CXCL11	C-X-C motif chemokine 11	Mann-Whitney	0.57
LAP TGF-beta-1	Latency-associated peptide transforming growth factor beta-1	Mann-Whitney	0.77
TRAIL	TNF-related apoptosis-inducing ligand	t-test	0.79
CDCP1	CUB domain-containing protein 1	t-test	0.92
CXCL1	C-X-C motif chemokine 1	Mann-Whitney	0.98

P-values in **bold** indicate statistical significance after Bonferroni correction (0.05/58=0.00086)
