# Supplementary material for: Posthemorrhagic hydrocephalus associates with elevated inflammation and CSF hypersecretion via activation of choroidal transporters
Source: Fluids Barriers CNS. 2022 Aug 10;19:62. doi: 10.1186/s12987-022-00360-w (PMC9367104; doi:10.1186/s12987-022-00360-w)
Supplement: Supplementary file 6 — Additional file 6: Table S4. Elevated inflammatory markers in CSF from PHH patients versus healthy control subjects. [file 12987_2022_360_MOESM6_ESM.pdf]

Additional file 6. Elevated inflammatory markers in CSF from PHH patients versus healthy control subjects.

**Marker**	**Full Name**	**Control subjects**	**PHH patients**	***P* value**
CCL3	C-C motif chemokine 3	4.46 ± 0.47	7.47 ± 1.78	<0.0001
CCL4	C-C motif chemokine 4	4.42 ± 0.37	8.32 ± 1.88	<0.0001
CCL20	C-C motif chemokine 20	3.63 ± 0.83	8.51 ± 2.08	<0.0001
IL-6	Interleukin-6	2.76 ± 0.71	8.51 ± 1.99	<0.0001
IL-10	Interleukin-10	1.74 ± 0.36	3.75 ± 1.09	<0.0001
LIF	Leukemia inhibitory factor	1.36 ± 0.40	7.40 ± 2.54	<0.0001
MCP-1	Monocyte chemotactic protein 1	12.74 ± 0.78	14.79 ± 0.44	<0.0001
OSM	Oncostatin-M	2.44 ± 0.51	6.35 ± 1.89	<0.0001
MCP-3	Monocyte chemotactic protein 3	1.34 ± 0.41	4.61 ± 2.17	<0.001
IL-8	Interleukin-8	8.20 ± 0.68	10.44 ± 1.59	<0.001

Data are expressed as Normalized Protein Expression (NPX) values (mean ± SD). Data were analyzed with an unpaired two-tailed t-test or a Mann-Whitney test and the Bonferroni correction was applied to accommodate multiple comparisons.
