# Supplementary material for: Posthemorrhagic hydrocephalus associates with elevated inflammation and CSF hypersecretion via activation of choroidal transporters
Source: Fluids Barriers CNS. 2022 Aug 10;19:62. doi: 10.1186/s12987-022-00360-w (PMC9367104; doi:10.1186/s12987-022-00360-w)
Supplement: Supplementary file 7 — Additional file 7: Table S5. Decreased inflammatory markers in CSF from PHH patients versus healthy control subjects. [file 12987_2022_360_MOESM7_ESM.pdf]

Additional file 7. Decreased inflammatory markers in CSF from PHH patients versus healthy control subjects.

**Marker**	**Full Name**	**Control subjects**	**PHH Patients**	***P* value**
CX3CL1	Fractalkine	2.72 ± 0.28	1.74 ± 0.51	<0.0001
DNER	Delta and Notch-like epidermal growth factor-related receptor	10.38 ± 0.06	9.53 ± 0.42	<0.0001
FGF-5	Fibroblast growth factor 5	3.90 ± 0.51	1.96 ± 0.64	<0.0001
Flt3L	Fms-related tyrosine kinase 3 ligand	9.23 ± 0.50	8.09 ± 0.61	<0.0001
IL-18	Interleukin-18	6.42 ± 0.85	4.45 ± 1.16	<0.0001
LIF-R	Leukemia inhibitory factor receptor	4.35 ± 0.45	3.05 ± 0.37	<0.0001
PD-L1	Programmed cell death 1 ligand 1	4.92 ± 0.46	3.43 ± 0.64	<0.0001
TWEAK	Tumor necrosis factor ligand superfamily, member 12	10.89 ± 0.52	8.16 ± 0.69	<0.0001
4E-BP1	Eukaryotic translation initiation factor 4E-binding protein 1	9.02 ± 1.66	4.53 ± 1.63	<0.0001
CSF-1	Macrophage colony-stimulating factor 1	9.73 ± 0.38	8.71 ± 0.50	<0.0001
ADA	Adenosine Deaminase	6.70 ± 0.96	4.87 ± 0.92	<0.001
ST1A1	Sulfotransferase 1A1	4.19 ± 1.10	2.47 ± 0.85	<0.001
AXIN1	Axin-1	2.53 ± 0.71	1.32 ± 0.62	<0.001
STAMBP	STAM-binding protein	5.01 ± 1.19	3.06 ± 1.04	<0.001

Data are expressed as Normalized Protein Expression (NPX) values (mean±SD). Data were analyzed with an unpaired two-tailed t-test or a Mann-Whitney test and the Bonferroni correction was applied to accommodate multiple comparisons.
